# Increasing inflammatory biomarkers are associated with mortality in critically ill COVID-19 patients despite anti-inflammatory treatment

**DOI:** 10.1007/s10238-025-01904-8

**Published:** 2025-11-11

**Authors:** Katrijn Daenen, Dimitris Rizopoulos, Virgil A. S. H. Dalm, Jilske A. Huijben, Sara C. M. Stoof, Nicole M. A. Nagtzaam, Willem A. Dik, Sigrid M. A. Swagemakers, Peter J. van der Spek, Kirby Tong-Minh, Daniel G. Aynekulu Mersha, Jessica Khyali, Nicole P. Juffermans, Diederik Gommers, Eric C. M. van Gorp, Lieuwe D. J. Bos, Henrik Endeman

**Affiliations:** 1 Department of Intensive Care, Erasmus University Medical Center Rotterdam, Doctor Molewaterplein 40, 3015 GD Rotterdam, The Netherlands; 2 Department of Viroscience, Erasmus University Medical Center Rotterdam, Doctor Molewaterplein 40, 3015 GD Rotterdam, The Netherlands; 3 Department of Biostatistics, Erasmus University Medical Center, Rotterdam, The Netherlands; 4 Department of Epidemiology, Erasmus University Medical Center, Rotterdam, The Netherlands; 5 Department of Immunology, Erasmus University Medical Center Rotterdam, Rotterdam, The Netherlands; 6 Department of Internal Medicine, Division of Allergy & Clinical Immunology, Erasmus University Medical Center, Rotterdam, The Netherlands; 7 Laboratory Medical Immunology, Department of Immunology, Erasmus University Medical Center, Rotterdam, The Netherlands; 8 Laboratory Medical Immunology, Reinier Haga Medisch Diagnostisch Centrum (RHMDC), Delft, The Netherlands; 9 Department of Pathology & Clinical Bioinformatics, Erasmus MC University Medical Center Rotterdam, Rotterdam, The Netherlands; 10 Laboratory of Translational Intensive Care, Erasmus MC, University Medical Center, Rotterdam, the Netherlands; 11 Department of Internal Medicine, Erasmus University Medical Center, Rotterdam, The Netherlands; 12 Department of Intensive Care, Location Academic Medical Centre, Amsterdam University Medical Centers, University of Amsterdam, Amsterdam, The Netherlands; 13 Laboratory of Experimental Intensive Care and Anesthesiology, Location Academic Medical Centre, Amsterdam University Medical Centers, University of Amsterdam, Amsterdam, The Netherlands; 14 Department of Intensive Care, OLVG, Amsterdam, The Netherlands

**Keywords:** COVID-19, Acute respiratory distress syndrome, Mortality, Biomarker, Intensive care unit, Joint model, Repeated measurements

## Abstract

Predicting mortality in COVID-19 ARDS may support ICU clinical decision-making. Biomarkers of innate immunity, coagulation, endothelial injury, and fibroproliferation have been studied as predictors. We aimed to examine whether trends in plasma biomarkers predict ICU mortality and to explore underlying biological processes through pathway analysis. Additionally, we explored whether HDS changes biomarker trajectories in COVID-19 ARDS. In this observational study, we included patients with COVID-19 ARDS admitted to the ICU of an academic hospital in Rotterdam between February 2020 and February 2022. In repeated plasma samples, 64 biomarkers were measured. Joint modeling assessed the association between biomarker changes and ICU mortality, adjusting for age, sex, BMI, and HDS. Protein–protein interaction and gene ontology enrichment analyses were performed using STRING, Cytoscape, and DAVID EASE. Biomarker trajectories were compared between HDS-treated and non-treated patients, adjusting for timing, SOFA score, and tocilizumab. One hundred and sixty-two patients were included and 43 died during ICU stay. A doubling in the values of 26 biomarkers over the next day was predictive of ICU mortality (HRs 0.16–8.56; q < 0.05). Gene ontology enrichment analysis identified 19 overrepresented biological processes (FDR ≤ 0.05), with highest fold enrichment for macrophage chemotaxis, negative regulation of bone resorption, and leukocyte cell–cell adhesion. Forty-eight patients received HDS at a median of 6 ICU days. HDS significantly changed the trajectories of four mortality-associated biomarkers: Albumin and lactoferrin decreased, while CRP and VEGF increased. In COVID-19 ARDS, repeated biomarker measurements demonstrate a systemic inflammatory state associated with mortality. HDS changed trends of several biomarkers, but did not reduce those associated with fatal outcomes. *Trial registration*: ClinicalTrials.gov NCT05403359; https://clinicaltrials.gov/ct2/show/NCT05403359

**Supplementary Information:**

The online version contains supplementary material available at 10.1007/s10238-025-01904-8.

## Introduction

Acute respiratory distress syndrome (ARDS) is a life-threatening condition characterized by acute onset of inflammatory hypoxemic respiratory failure, involving uncontrolled immune cell activation and cytokine release [1–4]. The emergence of Coronavirus disease-2019 (COVID-19) significantly increased the incidence of ARDS cases requiring intensive care unit (ICU) admission worldwide [5]. This necessitated rapid insights into the pathophysiology, prognostic factors, and therapeutic options for this novel etiology of ARDS.

Pre COVID-19 pandemic research has shown that higher plasma levels of inflammatory biomarkers are associated with a higher risk of mortality in ARDS [6–9]. Studies examining the immunological profile of COVID-19-related ARDS patients have also demonstrated elevated levels of immune-inflammatory markers in peripheral blood [10–12]. Similar to non-COVID-19 ARDS, the excessive release of cytokines in COVID-19 ARDS is strongly associated with the risk of multiple organ failure and mortality [13]. Consequently, biomarkers of the immunopathophysiology of COVID-19-related ARDS may serve as mortality predictors that potentially could guide patient care. Additionally, pulmonary thrombosis was associated with mortality in critically ill COVID-19 patients, and therefore, biomarkers of coagulation, endothelial damage, and fibroproliferation are also of interest [14–16]. To gain deeper insights into these complex immunopathological mechanisms, gene ontology enrichment can provide valuable information about the underlying biological processes and their interactions [17, 18].

Corticosteroids became first-line immunosuppressive therapy for patients with COVID-19 because of their anti-inflammatory effects, and have clearly demonstrated mortality reduction in patients receiving oxygen therapy or invasive mechanical ventilation [19, 20]. Treatment with high-dose corticosteroids (HDS) is considered in COVID-19 patients presenting with ARDS or experiencing progressive clinical deterioration despite the standard corticosteroid regimen of dexamethasone 6 mg up to 10 days, in line with the ATS guidelines for ARDS management [21]. HDS mitigate severe hyperinflammation [22–24], and a reduction in pro-inflammatory biomarkers may therefore be reflective of decreased risk for death.

The aim of this study was to investigate systemic inflammation in patients with COVID-19 ARDS by measuring a comprehensive set of protein biomarkers in plasma. This set included both established biomarkers used in standard care and novel or less extensively studied markers, systematically selected following expert panel review of COVID-19, ARDS, and sepsis literature to identify biomarkers with enhanced prognostic utility in the COVID-19 ARDS setting. Moreover, we aimed to explore the interactions of the mortality-associated biomarkers and to identify their shared biological pathways. We hypothesized that patients with COVID-19-related ARDS exhibit a persistent systemic pro-inflammatory response associated with increased mortality risk. Furthermore, we hypothesized that treatment with HDS is associated with a decrease in the concentrations of systemic biomarkers that correlate with mortality. By using repeated measurements, we captured the dynamic nature of systemic inflammation, enabling a nuanced analysis of disease progression and the effects of HDS.

## Methods

### Study design

We conducted an observational, single-center, longitudinal cohort study. Data were analyzed from patients with COVID-19 ARDS admitted to the Erasmus University Medical Center (Rotterdam, the Netherlands) between February 22, 2020, and January 26, 2022. Patients were prospectively included as soon as possible after ICU admission, if they were at least 18 years of age and had either a polymerase chain reaction (PCR)-confirmed SARS-CoV-2 infection or high clinical suspicion of COVID-19. ARDS was defined according to the Berlin criteria [25]. Standard ICU care during the study period included selective digestive decontamination (SDD), which was initiated at the time of endotracheal intubation, and prophylactic anticoagulation with dalteparin, administered from the moment of ICU admission, with dosage adjustments made over the course of the pandemic. Sedation was provided with either midazolam and sufentanil or propofol, depending on availability. Neuromuscular blockade with rocuronium was used when prone positioning was indicated, which was applied in patients with a PaO₂/FiO₂ ratio below 150 mmHg after adequate PEEP settings had been established.

The study was approved by the local Medical Ethics Review Committee under protocol number MEC-2017–417 and conducted in accordance with the principles of the Declaration of Helsinki. Due to the urgency of conducting research in patients with COVID-19, an exemption for consent was approved by the Medical Research Ethics Committee at the Erasmus University Medical center. An opt-out informed consent procedure (MEC-2022–0297) was used, and patients who expressed objections to participation were excluded from the study. No other exclusion criteria were applied.

### Data collection, sampling, and assays

Patient data, including demographics, body mass index (BMI), vital signs, comorbidities, APACHE-IV, SOFA score, and laboratory tests, were extracted from the electronic health record upon ICU admission and were collected during follow-up. Malignancy was recorded as a comorbidity in cases of both solid organ tumors and hematological malignancies. Immunodeficiency was recorded as a comorbidity in patients with a documented primary immunodeficiency, or a secondary immunodeficiency due to conditions such as HIV infection, hematological or solid malignancies, use of immunosuppressive therapy, or asplenia. The PaO₂/FiO₂ (P/F) ratios were calculated using the PaO₂ and FiO₂ measurements collected closest to 8 AM on the same day. Blood samples were collected at 06.00 AM from all included patients for laboratory testing on days 0,1,2, 7, 14, 21, and 28, as long as patients were admitted at the ICU.

Based on expert consensus from an interdisciplinary panel of immunologists, laboratory specialists, intensivists, and methodologists, and following literature review, 54 biomarkers indicative of key pathophysiological pathways in COVID-19 ARDS were selected—including biomarkers of (pro)inflammation, coagulation, fibroproliferation, epithelial and endothelial damage or dysfunction, and growth factors—and analyzed using a Luminex assay (Table S1). In addition, ten routine care biomarkers were obtained from the electronic health record, measured on the same days as the aforementioned samples. These markers included C-reactive protein (CRP), D-dimer, ferritin, interleukin-6 (IL-6), lactate dehydrogenase (LDH), neutrophil-to-lymphocyte ratio (NLR), procalcitonin (PCT), albumin, alanine aminotransferase (ALAT) and leukocyte count.

### Study exposure and outcome

 HDS was defined as treatment with > 6 mg of dexamethasone per day or an equivalent dose of another corticosteroid administered for at least 1 day (Table S2). HDS could be administered at any time from hospital admission until discharge from ICU and thus was considered a time-updated dependent exposure. At the beginning of the pandemic, the decision to start HDS was made based on clinical judgment and multidisciplinary discussion. From April 7, 2021, protocols were established at the Erasmus University Medical Center, which were largely based on existing ARDS guidelines (Supplementary file 2) [22]. The protocol recommended HDS for patients diagnosed with moderate COVID-19 (stage IIb), characterized by pulmonary involvement with hypoxemia, or severe COVID-19 (stage III) with additional extrapulmonary systemic hyperinflammation, if diagnosis occurred within 14 days of symptom onset. Treatment consisted of 1000 mg methylprednisolone administered for 1 to 3 days. The distinction between stages was based on respiratory parameters, imaging findings, and systemic inflammation assessed through inflammatory markers. Patients met the criteria for stage IIb or III if they had a PaO₂ /FiO₂ ratio < 300 mmHg or compliance < 60 mL/cmH2O (Table S3) [26]. The primary outcome for analysis was all-cause ICU mortality. Patients were monitored from ICU admission until ICU discharge.

### Statistical analysis

Normally distributed baseline variables were reported as mean with standard deviation (SD), and non-normally distributed variables as median with interquartile range (IQR). Differences in categorical variables between survivors and non-survivors were analyzed using chi-square tests, with Fisher's exact tests used for variables with low cell frequencies. Continuous variables were compared using an independent samples t test for normally distributed data and a Mann–Whitney U test for non-normally distributed data. For age, sex, body mass index (BMI), and HDS, standard Cox regression model analysis was presented. Kaplan–Meier curves were presented for the survival function, including censoring at ICU discharge.

Temporal changes in absolute plasma biomarker concentrations and the association between the biomarkers and the risk of all-cause-mortality in the ICU were assessed using a joint model that combines a linear mixed effect model and a Cox proportional hazards model. All the linear mixed effects and Cox sub-models in all joint models were adjusted for age, sex, BMI, and the use of HDS at sampling time. To address the limits of detection and skewed distributions of many biomarkers, we applied logarithmic transformation (base 2) and used linear mixed models that account for censoring. This means that the reported hazard ratios (HRs) express the relative change in the risk of death resulting from a doubling of the biomarker’s value in a day compared to no change in the same period. Splines were incorporated into both the fixed and random effects parts for biomarkers with nonlinear shapes of the patient-specific longitudinal trajectories. Proportional hazards assumptions were evaluated by allowing the coefficients for the markers to be time-varying and comparing these with the time-constant coefficients; no serious violations of this assumption were detected. The linear assumption for the effect of biomarkers on the log hazard scale was assessed by incorporating polynomial terms and evaluating their statistical significance; no important deviations from linearity were identified.

The network of biomarkers significantly associated with mortality in the joint model analyses was visualized using STRING database (Search Tool for the Retrieval of Interacting Genes/Proteins) [27, 28] and Cytoscape software [29], which provides known and predicted protein–protein interactions. For functional pathway analysis, these biomarkers underwent gene ontology enrichment analysis using DAVID (Database for Annotation, Visualization, and Integrated Discovery), utilizing all Homo sapiens genes as a reference [30, 31]. A significance threshold of FDR ≤ 0.05 was set. Because HDS treatment is known to mitigate (hyper)inflammation, we studied the association between HDS and changes in systemic biomarker concentrations. This analysis was solely performed on the biomarkers that were significantly associated with ICU mortality, as identified by joint modeling. Biomarker trajectories were compared between patients who did and did not receive HDS by using linear mixed effects models (*lme*4 package) at several time points (day 0, 2, 3, and 7). The model was adjusted for SOFA score, timing of HDS, and tocilizumab administration. Statistical analyses were performed using R version 4.4.3 using the JMbayes2 package (10.32614/CRAN.package.JMbayes2). Statistical significance was set at p < 0.05, and p-values were adjusted for multiple testing using the false discovery rate method.

## Results

One hundred and sixty-two patients with COVID-19 related ARDS were included between February 22, 2020, and January26, 2022. In total, 47 (29%) patients died during ICU stay. A total of 156 samples were collected on day 0 of inclusion, followed by 148 samples on day 1, 127 samples on day 2, 114 samples on day 7, 78 samples on day 14, 49 samples on day 21, and 26 samples on day 28 (Figure S1). Overall, the median age was 64 years (IQR = 56–70), median BMI was 28.4 kg/m^2^ (IQR = 25.7–32.4), and 77% of the patients were male (Table 1). Non-survivors were significantly older (p = 0.003) and had a lower BMI (p = 0.037) than survivors. Moreover, APACHE-IV and SOFA scores were higher (p < 0.001) and PaO₂/FiO₂ ratios were lower (p = 0.015) in non-survivors, indicating greater disease severity at baseline. There were no significant differences in the proportion of patients with various comorbidities. Tocilizumab was administered to 40 (25%) patients and dexamethasone 6mg to 76 (47%) patients, with no significant difference observed between survivors and non-survivors. The Kaplan–Meier curve for survival is shown in Figure S2.

**Table 1:** Demographic and clinical characteristics at baseline

	All patients N = 162	Survivors N = 119	Non-survivors N = 43	P value
*Patients’* *characteristics*				
Male, (N, %)	125/162 (77%)	87/119 (73%)	38/43 (88%)	0.067
Age in years	64 (56–70)	62 (53- 69)	67 (62- 72)	0.003
BMI in kg/m^2^	28.4 (25.7,-32.4)	29.4 (26.4,-32.4)	26.5 (24.8- 31.9)	0.037
*Comorbidities,* *(N,* *%)*				
Hypertension	51/116 (44%)	38/87 (44%)	13/29 (45%)	> 0.99
Peripheral vascular disease	5/118 (4.2%)	3/88 (3.4%)	2/30 (6.7%)	0.60
CVA/TIA	6/118 (5.1%)	4/88 (4.5%)	2/30 (6.7%)	0.60
Chronic pulmonary disease	11/104 (9.6%)	8/86 (9.3%)	3/28 (11%)	> 0.99
Chronic kidney disease	9/118 (7.6%)	4/84 (4.5%)	5/30 (17%)	0.05
Immunodeficiency	9/118 (7.8%)	2/30 (6.7%)	7/86 (8.1%)	> 0.99
Malignancies	10/118 (8.5%)	8/88 (9.1%)	2/30 (6.7%)	> 0.99
Diabetes mellitus	23/118 (19%)	8/30 (27%)	15/88 (17%)	0.30
*Admission* *characteristics*				
APACHE-IV, %*	20% (10%-20%)	20% (10%-30%)	30% (20%-40%)	0.001
SOFA score	6.0 (5.0, 9.0)	6.0 (4.0, 7.8)	7.0 (6.0, 11.0)	< 0.001
PaO₂/FiO₂ ratio, mmHg	210 (143, 278)	225 (150, 285)	180 (135, 233	0.015
*Laboratory* *parameters*				
C-reactive protein, mg/L	106 (15, 301)	71 (12, 296)	179 (27, 314)	0.30
D-dimer, mg/L	1.8 (1.0, 4.0)	1.6 (0.9, 3.7)	1.9 (1.3, 5.2)	0.13
Lactate dehydrogenase, U/L	328 (272, 413)	323 (270, 394)	355 (295, 487)	0.029
Ferritin, ug/L	1,258 (779, 1,859)	1,275 (730, 1,839)	1,213 (958, 2,042)	0.40
Procalcitonin, ng/mL	0.4 (0.1, 1.2)	0.3 (0.1, 1.0)	0.7 (0.2, 1.6)	0.013
Alanine aminotransferase, U/L	49 (28, 81)	49 (29, 81)	54 (24, 76)	0.80
Interleukin-6, pg/L	122 (43, 295)	113 (33, 246)	201 (85, 458)	0.017
Albumin, g/L	21.0 (17.5, 23.0)	21.0 (18.0, 24.0)	20.0 (17.0, 22.0)	0.13
Table 1 Continued				
Leukocytes, × 10^9^	9.6 (7.4, 12.3)	9.5 (7.4, 11.9)	10.5 (8.0, 13.3)	0.30
Neutrophil-to-Lymphocyte ratio	9 (5, 14)	7 (5, 11)	17 (11, 23)	< 0.001
*Medication* *administered* *during* *admission,* *(N,* *%)*				
High-dose corticosteroid therapy**	52 (32%)	28 (24%)	24 (56%)	< 0.001
Tocilizumab	40 (25%)	29 (24%)	11 (26%)	> 0.99
Dexamethasone 6mg	76 (47%)	55 (46%)	21 (49%)	> 0.99
*Clinical* *outcomes*				
ICU length of stay in days	17 (9–28)	16 (9–28)	19 (10–25)	> 0.99

All continuous variables are reported as median [IQR] and all categorical variables as counts and percentages. Survivors and non-survivors were compared using a Wilcoxon test for continuous variables and Pearson’s χ2 test for categorical variables

^*^ Estimated Mortality Rate APACHE-IV Score in percentage **High-dose corticosteroid therapy was defined as treatment with > 6 mg of dexamethasone per day or equivalent

There were missing data for BMI in two patients (1.2%), for SOFA score in 11 patients (6.8%), for P/F ratio in 16 patients (9.9%), and for APACHE-IVscore in 40 patients (24.7%). Abbreviations: APACHE, acute physiology, age, and chronic health evaluation; ARDS, acute respiratory distress syndrome; BMI, body mass index; FiO₂, fraction of inspired oxygen; NLR, neutrophil-to-lymphocyte ratio; PaO₂, arterial oxygen pressure; SOFA, sequential organ failure assessment

### Plasma inflammatory response over time

Longitudinal inflammatory responses were assessed using repeated measurements to evaluate biomarker trajectories. After quality assessment, biomarkers IFN-β and G-CSF were considered unreliable and therefore excluded from the analysis (Table S4). The joint model analysis revealed significant changes in various (pro)inflammatory biomarkers over time, including lactoferrin, CCL2, CXCL10, GM-CSF, IL-6, IL-10, IL-18, IL-7, TNF-α, osteoprotegerin, IL-6Rα, CXCL16, C5a, IL-33, IFN-α, IFN-γ, IL-28, IL-1 RI, CCL20, LEP, and IL-8. Additionally, significant changes over time were observed in endothelial markers VCAM-1, Tie-2, E-selectin, fibroproliferative markers galectin-3, MMP-9, the epithelial marker RAGE, coagulation Factor III, and the pro-angiogenic growth factor VEGF (Table S5). Furthermore, standard care markers that showed significant changes over time included CRP, ferritin, leukocyte count, ALAT, LDH, NLR, albumin, d-dimer, and procalcitonin (Table S5). The concentrations of the other biomarkers remained relatively stable throughout the observation period. The trajectories of all biomarkers significantly associated with mortality are visualized in Figure S3, illustrating the direction and magnitude of changes over time.

### Association between biomarker levels and mortality

**Fig. 1:**
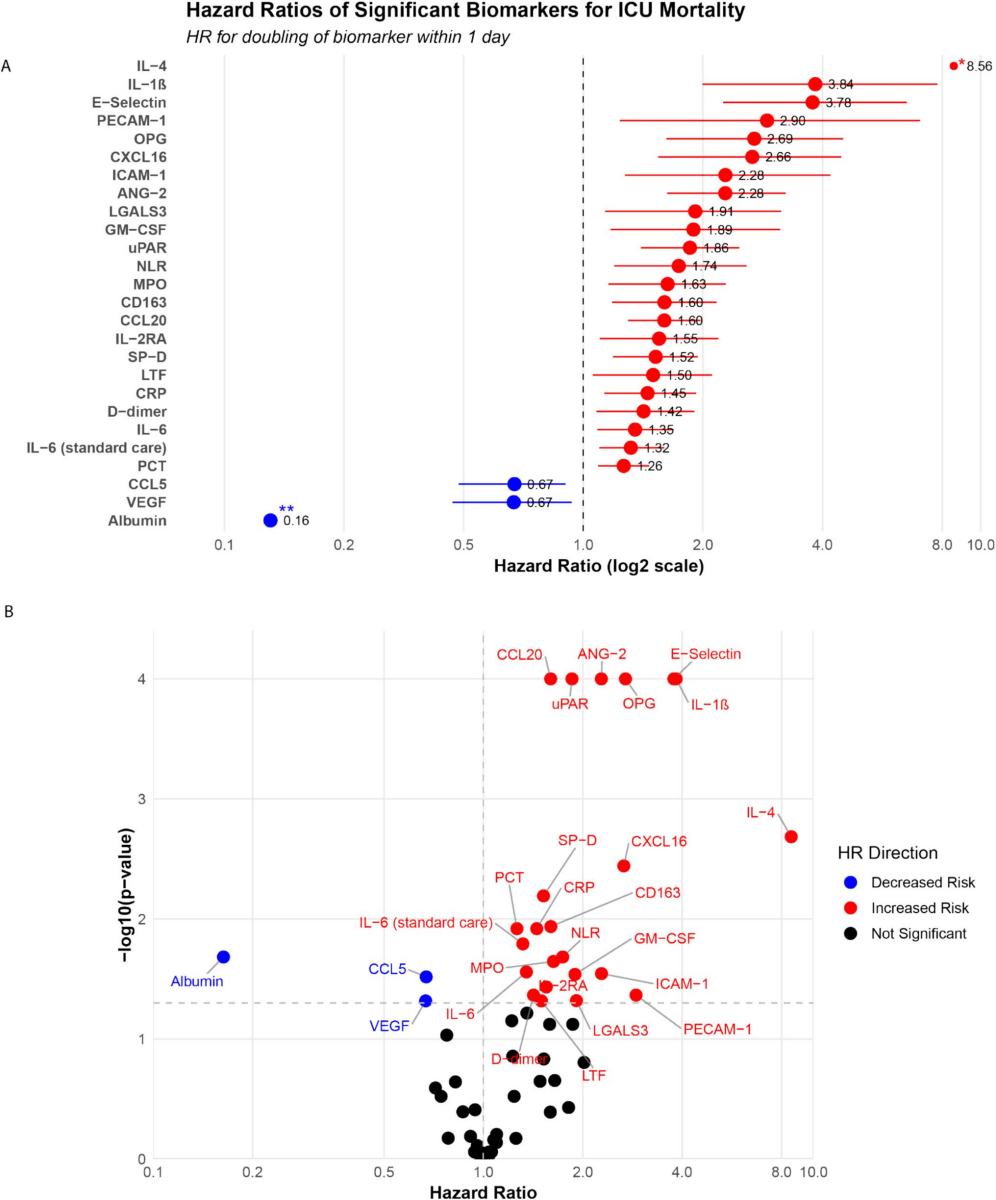
Relationship between hazard ratios and statistical significance for biomarkers associated with ICU mortality. In both panels, the x-axis shows hazard ratios on a logarithmic scale, representing the change in mortality risk for each doubling of biomarker concentration. The horizontal dashed line indicates the significance threshold (p = 0.05) after adjustment for multiple testing using the False Discovery Rate method. **A** Forest plot showing significant biomarkers ordered by hazard ratio magnitude. Red dots indicate increased mortality risk; blue dots indicate decreased risk. Horizontal lines represent 95% confidence intervals. *IL-4 confidence interval = 2.93–27.46; **Albumin confidence interval = 0.04–0.59 **B** Volcano plot displaying all tested biomarkers. The y-axis shows log10 (p-value), with higher values indicating greater statistical significance. Red dots indicate biomarkers significantly associated with increased mortality risk; blue dots indicate significantly decreased risk; black dots represent non-significant associations. All biomarker names and their abbreviations are provided in Table S1

After adjusting for age, gender, BMI, HDS, and multiple testing, 26 biomarkers emerged as significant predictors of ICU mortality (Table S6). The HRs for all biomarkers and the covariates are presented in Table S7. For albumin, VEGF, and CCL5, a doubling of the biomarker level resulted in a decreased hazard ratio for mortality, while for all other significant predictive biomarkers, this resulted in an increased hazard ratio. Among these, IL-4, IL-1β, and albumin showed the most extreme HRs for the association with ICU mortality, with HRs of 8.56 (95% CI: 2.93–27.46), 3.84 (95% CI: 2.00–7.79), and 0.16 (95% CI: 0.043–0.59), respectively (Fig. 1). Six of those 26 significant biomarkers were routinely assessed as part of standard care, including IL-6, procalcitonin, D-dimer, albumin, CRP, and NLR.

Fig. 1 Biomarker associations with ICU mortality.

### Pathway analysis of biomarkers significantly associated with IC mortality

**Fig. 2:**
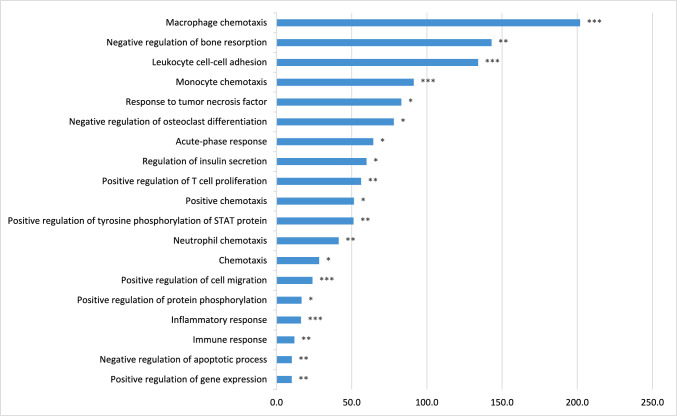
GO enrichment analysis of biomarkers significantly associated with ICU mortality. The enriched GO terms for biological processes related to all 26 selected biomarkers are visualized. Enriched categories are those identified as significantly overrepresented based on the EASE score (x-axis), which employs a modified Fisher's exact probability test. Statistical significance is indicated as follows: *, FDR < 0.05; **, FDR < 0.01; ***, FDR < 0.001

The network of significant biomarkers was visualized using STRING database and Cytoscape software, as shown in Figures S4 and S5. In both, IL-6 was identified as central hub and key mediator within the pathophysiological network, showing the highest degree of betweenness centrality among all the markers analyzed. Subsequently, the 26 significant biomarkers were used for GO enrichment analysis, which pointed to 19 biological processes that were overrepresented (FDR ≤ 0.05) (Table S8). This analysis revealed that the biomarkers that were significantly associated with ICU mortality were primarily mapped to macrophage, monocyte and neutrophil chemotaxis, negative regulation of bone resorption and osteoclast differentiation, negative regulation of apoptotic process, leukocyte cell–cell adhesion, T-cell proliferation, regulation of tyrosine phosphorylation of STAT protein, cell migration, and cytokine production (Fig. 2).

Fig. 2 Biological. processes associated with ICU mortality.

### Association between HDS treatment and biomarker levels

**Fig. 3:**
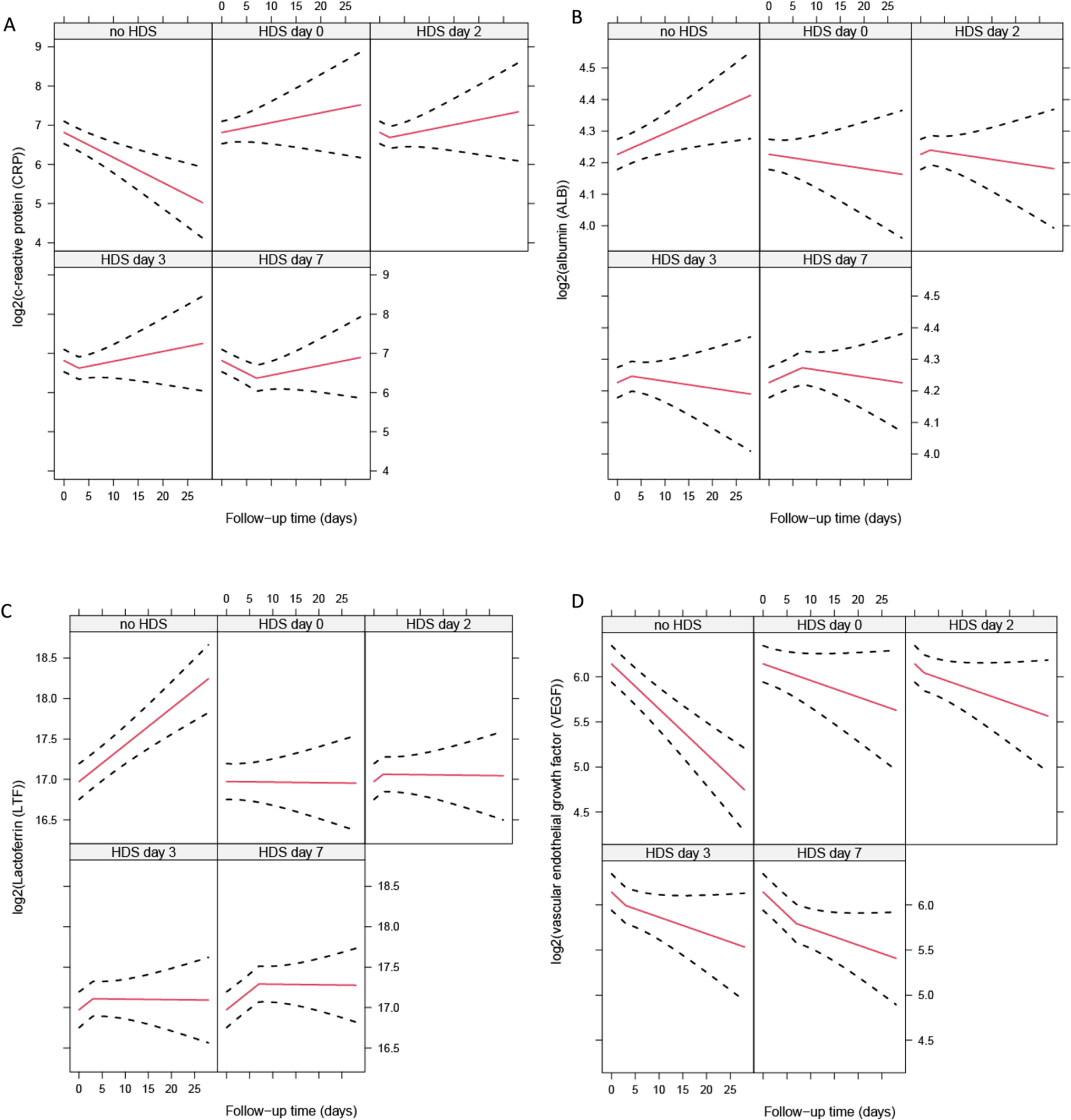
Trajectories of biomarker levels in patients treated with HDS (n = 48) and without HDS therapy (n = 114). The figure shows the trajectory of log₂-transformed biomarker levels (y-axis) versus follow-up time in days (x-axis) for different time points. Red lines represent the mean predicted values with 95% confidence intervals shown as dashed black lines. The panels display the temporal changes in biomarkers when not treated with HDS, at HDS initiation at day 0, and at subsequent timepoints days 2, 3, and 7: Fig. 3A shows albumin (ALB) levels, Fig. 3B shows C-reactive protein (CRP) levels, Fig. 3C shows lactoferrin (LTF) levels, and Fig. 3D shows vascular endothelial growth factor (VEGF) levels

Forty-eight patients (30%) received HDS at a median of 6 days after ICU admission, and baseline characteristics stratified on patients who did or did not receive HDS during their ICU admission are presented in Table S9. Forty-five out of the 48 patients treated with HDS received a standardized 3-day course of 1000 mg methylprednisolone followed by a prednisolone tapering regimen (starting at 1 mg/kg with a maximum of 100 mg, reduced by 10 mg weekly). Using linear mixed effects models, the trajectories of the biomarkers were compared between patients who did and did not receive HDS at several time points. HDS was significantly associated with change in the slope for RAGE (*p* = 0.0002), CRP (*p* = 0.0013), lactoferrin (*p* = 0.0023), CCL2 (*p* = 0.0089), IL-1RI (*p* = 0.0096), IL-18 (*p* = 0.01), C9 (*p* = 0.02), Tie-2 (*p* = 0.02), coagulation factor III (*p* = 0.02), CXCL10 (*p* = 0.02), VEGF (*p* = 0.04), leukocyte count (*p *= 0.04), and albumin (*p* = 0.05). Among the 26 aforementioned biomarkers that were significantly associated with ICU mortality, patients treated with HDS showed a significant change in the slope for the biomarkers albumin (*p* = 0.05), CRP (*p* = 0.001), lactoferrin (*p* = 0.0009) and VEGF (*p* = 0.03). HDS-treated patients were found to have decreasing slopes of albumin and lactoferrin after treatment initiation, whereas CRP and VEGF showed increasing slopes after HDS initiation. The effect plots in Fig. 3 visualize the effect of initiating HDS on days 0, 2, 3, and 7 versus never initiating the medication. The longitudinal analysis of the other biomarkers significantly associated with ICU mortality revealed no differences between the HDS-treated and untreated groups.

Fig. 3. Effect of HDS on biomarker trajectories associated with ICU mortality.

## Discussion

This study identified 26 biomarkers that were significantly associated with ICU mortality in COVID-19 ARDS, with the majority involved in innate immune responses, some in the adaptive immune responses, and a few involved in endothelial, coagulation, or fibroproliferative processes. An upward trend in the concentrations of these biomarkers was associated with increased mortality risk, except for albumin, CCL5, and VEGF, where a downward trend in concentrations was associated with an increased mortality risk. These findings support the concept of a systemic hyperinflammatory state as a key contributor to poor outcomes. Gene ontology analysis of the mortality-associated biomarkers identified macrophage and monocyte chemotaxis, negative regulation of bone resorption, and leukocyte cell–cell adhesion as the most overrepresented biological processes, suggesting that alterations in these processes contribute to poor outcomes. Interestingly, only albumin, lactoferrin, CRP, and VEGF were both associated with mortality and exhibited significant changes in slope under the influence of HDS treatment. Specifically, albumin and lactoferrin showed decreasing trends, whereas CRP and VEGF showed increasing trends.

In this study, we observed time-dependent changes in the systemic inflammatory response, demonstrating that an elevated inflammatory response was associated with increased mortality risk in our COVID-19 ARDS cohort. This finding is consistent with the established relationship between persistent hyperinflammation and higher mortality in non-COVID-19 ARDS and with similar observations in other COVID-19 ARDS cohorts [6, 32–37]. The association was most pronounced for biomarkers indicative of innate immunity, including IL-1β, CCL20, CCL5, CRP, CD163, IL-6, lactoferrin, ICAM-1, MPO, uPAR, GM-CSF, procalcitonin, NLR, and galectin-3. The predominance of innate immune markers may reflect the timing of our study, as our cohort primarily included patients from the early pandemic waves when SARS-CoV-2 was a novel pathogen and the population had no preexisting immunity. We propose that the immediate, non-specific innate immune response during early COVID-19 infection played a central role in the hyperinflammatory state associated with poor outcomes in critically ill COVID-19 patients. Among these biomarkers, IL-1β demonstrated the strongest association with mortality. IL-1β is produced following activation of the innate immune system and plays a pivotal role in the cytokine storm [38–40]. Prior research consistently supports an association between elevated IL-1β levels and increased mortality in COVID-19 [41–43].

While the innate immune response appeared to dominate the early inflammatory profile, several biomarkers indicative of adaptive immunity, including IL-4, IL-2Rα, CXCL16, and osteoprotegerin, were also associated with mortality. Notably, IL-4, a key player in adaptive immune responses through its promotion of T helper 2 (Th2) cell differentiation, B-cell proliferation, and IgE production, showed a particularly strong association with poor outcomes [44, 45]. This finding aligns with a meta-analysis demonstrating that elevated IL-4 levels are associated with increased COVID-19 severity and mortality, with significantly higher concentrations observed in non-survivors compared to survivors [46, 47]. The known involvement of IL-4 in fibrotic pathways and impaired viral clearance could represent potential mechanisms linking elevated IL-4 levels with poor prognosis in COVID-19 [48, 49]. Furthermore, our findings revealed that biomarkers reflecting fibroproliferative processes (VEGF, uPAR, galectin-3, E-selectin, and surfactant protein D), endothelial dysfunction (ANG-2, PECAM-1, and ICAM-1), and D-dimer were also associated with increased mortality risk. These results suggest that beyond the inflammatory cascade, both endothelial damage, coagulation, and dysregulated tissue repair mechanisms are relevant processes contributing to poor outcomes in COVID-19 ARDS. This observation is consistent with the high incidence of pulmonary thrombosis observed in critically ill COVID-19 patients that was associated with mortality during the pandemic [15, 16, 50, 51].

Albumin, CCL5, and VEGF showed inverse associations with mortality compared to the other biomarkers in our study. The relationship between lower albumin concentrations and higher mortality is well-established, as albumin is a negative acute-phase protein that decreases during severe inflammation [32, 52–54]. Interestingly, our findings regarding VEGF diverge from the predominant literature on ARDS, including COVID-19, which typically reports elevated VEGF levels associated with poor outcomes [55–57]. Our findings might suggest a potential impairment of vascular repair mechanisms during severe disease contributing to poor outcomes. The observation that lower CCL5 levels predict worse outcomes challenges the hyperinflammatory state hypothesis of COVID-19 mortality. While the literature on CCL5 in COVID-19 remains inconclusive, several studies have documented reduced CCL5 levels in critically ill patients compared to those with mild disease [58–60]. This pattern suggests that CCL5 reflects a protective function rather than contributing to pathology in critically ill COVID-19 patients. An alternative explanation for these findings is that lower CCL5 levels may reflect T-cell exhaustion, consistent with emerging evidence of a biphasic immune trajectory with early hyperinflammation followed by immunoparalysis and relative immunosuppression [61, 62].

Gene ontology enrichment analysis identified 19 overrepresented biological processes among our mortality-predictive biomarkers, offering insights into the pathophysiological pathways underlying COVID-19 ARDS and potentially serving as foundation for identifying potential therapeutic targets. The highest fold enrichment was observed in macrophage and monocyte chemotaxis, negative regulation of bone resorption, and leukocyte cell–cell adhesion. The prominence of chemotaxis and adhesion processes reflects the systemic innate inflammatory response in severe COVID-19, thereby supporting the hypothesis that the hyperinflammatory response of the innate immune system is a key driver of poor outcomes. This aligns with other evidence suggesting that heightened and sustained innate immune activation in severe COVID-19 leads to delayed adaptive immune responses, resulting in excessive tissue damage and unfavorable outcomes [63]. Also noteworthy was the overrepresentation of negative regulation of bone resorption, which might be a response to the elevated IL-4 levels in our cohort. Namely, IL-4 inhibits bone resorption by increasing osteoprotegerin expression, which blocks RANKL-RANK binding and subsequent osteoclast activation [64–66]. The significant association of osteoprotegerin with IC mortality in our cohort suggests this pathway's importance in critically ill COVID-19 patients. However, these findings primarily highlight biological processes that are not unique to COVID-19 but are frequently observed across a range of severe diseases, warranting further validation to determine their clinical relevance [67–71].

HDS was administered based on the hypothesis that reducing systemic inflammation would benefit patients with COVID-19 ARDS [19, 39, 72, 73]. To evaluate this assumption, we investigated the association between HDS treatment and biomarker trends. Among the biomarkers significantly associated with mortality, HDS treatment was linked to significant changes in only four markers: decreased concentrations of albumin and lactoferrin, and increased CRP and VEGF concentrations. These findings provide limited support for the hypothesis that HDS effectively targets the persistent systemic inflammatory state in COVID-19 ARDS. The reduction in lactoferrin, an important antimicrobial and anti-inflammatory protein [74], and the fact that increased lactoferrin levels were associated with increased mortality in our cohort, is the only finding supporting our hypothesis that HDS might reduce mortality by reducing inflammation. Conversely, the observed increase in CRP contradicts the expected anti-inflammatory effect of HDS. This discrepancy may be explained by the observational nature of our study; despite corrections for indication bias, unmeasured residual confounding likely persisted. Thus, increasing CRP and decreasing albumin levels may have led to HDS administration rather than resulting from it. Alternatively, these findings regarding CRP could reflect (bacterial) superinfections making HDS not the appropriate treatment, or be a consequence of the immunosuppressive side effects of steroids increasing susceptibility to secondary infections and therefore increasing the risk of poor outcomes [75, 76]. Additionally, the increase in VEGF—a key player in angiogenesis and vascular permeability—following HDS treatment suggests that steroids may modulate the endothelial response in a potentially detrimental manner, as elevated VEGF levels correlated with increased mortality. To conclusively determine the causal relationships between HDS treatment, biomarker dynamics, and mortality outcomes, randomized controlled trials are necessary.

A limitation of our study is that we can only demonstrate associations between biomarkers and outcomes, without establishing causative relationships. Given that our study investigated a selected biomarker panel rather than unbiased biomarker discovery, our biological process analysis results are inherently dependent on the initial biomarker panel composition, potentially limiting the discovery of other relevant pathophysiological mechanisms. Furthermore, HDS treatment was not randomly assigned but initially determined by physician discretion and later by protocols based on specific patient characteristics, introducing potential indication bias. Although we corrected for differences in disease severity and timing, residual indication bias probably exists. While this limitation is common across studies from this period, it warrants consideration when interpreting our results. In addition, our analysis compares patients receiving HDS with those not receiving HDS at that moment in time, which may influence biomarker interpretation as corticosteroid effects may be dose-dependent or cumulative. Future studies, for instance in other ARDS populations, could address this limitation through randomized dose-comparison designs in corticosteroid-naïve patients and through detailed analysis of cumulative corticosteroid exposure.” Despite these limitations, a key strength of our study is the use of a comprehensive biomarker panel in an ARDS population with a uniform etiology, providing valuable insights into the disease's pathophysiology. Another strength lies in the application of a joint modeling approach, which enabled robust analysis of repeated measurements, accounting for the entire ICU admission course and changes over time.

Our findings have several important implications. We demonstrated that accessible biomarkers are strongly associated with mortality risk, as six of the 26 significant biomarkers—such as CRP, procalcitonin, and NLR—are routinely measured in clinical care. Their widespread availability and cost-effectiveness offer practical advantages for implementation in guiding therapy, particularly in resource-limited settings. Among the non-routine biomarkers, IL-4 and IL-1β showed the strongest associations with mortality. IL-1β emerged as the most promising predictive biomarker, given its clinical availability, narrow confidence interval, and the existence of targeted therapies such as anakinra. Supporting this, results from the SAVE-MORE trial [77] demonstrated a significant reduction in mortality with anakinra treatment in COVID-19 patients, particularly when administered early in the disease course, before the onset of ARDS. Moving toward precision medicine, our data suggest that elevated IL-1β levels could be used to identify patients most likely to benefit from anakinra. Furthermore, our gene ontology analysis reinforces the relevance of IL-1β–associated pathways and may serve as an exploratory foundation for identifying additional therapeutic targets. Finally, the observed associations between HDS and inflammatory biomarkers do not support the hypothesis that HDS effectively reduces inflammation linked to mortality in COVID-19–associated ARDS. Future studies may benefit from more explicit consideration of dose-dependency, cumulative exposure and treatment duration when investigating biomarker dynamics in relation to corticosteroid therapy.

## Conclusion

In conclusion, an absolute doubling of plasma concentrations over a 24-h period at any point during ICU admission for several biomarkers including IL-4, IL-1β, E-Selectin, CD31, osteoprotegerin, CXCL16, ICAM-1, ANG-2, galectin-3, GM-CSF, uPAR, NLR, MPO, CD163, CCL20, IL-2RA, SP-D, lactoferrin, CRP, D-dimer, IL-6, and procalcitonin was significantly associated with increased ICU mortality. An absolute doubling of plasma concentrations of albumin, CCL5, and VEGF was associated with decreased ICU mortality. Patients with COVID-19 ARDS exhibited a systemic inflammatory state characterized by macrophage and monocyte activation, negative regulation of bone resorption, and leukocyte cell–cell adhesion and interferon pathway involvement, which was associated with increased mortality risk. Treatment with HDS was associated with a significant decrease in the concentrations of biomarkers albumin and lactoferrin, and increase in CRP and VEGF concentrations. These findings enhance the understanding of the complex immunopathology underlying COVID-19 ARDS.

## Supplementary Information

Below is the link to the electronic supplementary material.

Supplementary file1 (DOCX 1714 KB)

## Data Availability

The datasets used and analyzed during the current study are available from the corresponding author upon reasonable request.

## Abbreviations

APACHE: Acute physiology, age, and chronic health evaluation
ARDS: Acute respiratory distress syndrome
BMI: Body mass index
CI: Confidence intervals
COVID-19: Coronavirus disease-2019
CRP: C-reactive protein
HDS: High-dose corticosteroids
ICU: Intensive care unit
IL: Interleukin
LDH: Lactate dehydrogenase
PCT: Procalcitonin
P/F: PaO₂/FiO₂
