# Supplementary material for: Increasing inflammatory biomarkers are associated with mortality in critically ill COVID-19 patients despite anti-inflammatory treatment
Source: Clin Exp Med. 2025 Nov 11;25(1):361. doi: 10.1007/s10238-025-01904-8 (PMC12605395; doi:10.1007/s10238-025-01904-8)
Supplement: Supplementary file 1 — Supplementary file1 (DOCX 1714 KB) [file 10238_2025_1904_MOESM1_ESM.docx]

**Supplementary file**

# **Increasing inflammatory biomarkers are associated with mortality in critically ill COVID-19 patients despite anti-inflammatory treatment**

Katrijn Daenen, MD^1,2^, Dimitris Rizopoulos, PhD^3,4^, Virgil A.S.H. Dalm, MD, PhD^5,6,^, Jilske A. Huijben, MD, PhD^1^, Sara C. M. Stoof, MD, PhD^1^, Nicole M A Nagtzaam^7^, Willem A. Dik, PhD^7,8^, Sigrid M. A. Swagemakers^9^, Peter J. van de Spek, MD, PhD^9^, Kirby Tong-Minh, MD, PhD^1,2^, Daniel G. Aynekulu Mersha, MD^,1,2^, Jessica Khyali, MD^1^, Nicole P. Juffermans, MD, PhD^1,10^, Diederik Gommers, MD, PhD^1^, Eric C. M. van Gorp, MD, PhD^2,11^, Lieuwe D.J. Bos, MD, PhD ^12,13‡^, Henrik Endeman, MD, PhD^1,14‡^

**Supplementary methods**

Assays

Samples were centrifuged (3000 N Relative centrifugal force (Rcf)) at room temperature for 5 minutes and were aliquoted and stored at –80°C. The Luminex multiplex bead immunoassay (R&D Systems, Abingdon, UK) was performed according to the manufacturer’s protocol, with data acquisition on a Luminex MAGPIX machine. Results were reported in pg/mL, with quality assessed based on bead count and values outside the calibration curve limits (Table S2).
The routine care biomarkers were measured using standard laboratory assays during the follow-up period in the clinical chemistry laboratory of the Erasmus Medical Center using the Roche Cobas 8000 system (Roche Diagnostics, Rotterdam, the Netherlands). CRP and D-dimer levels were measured using a turbidimetric method (C502 Cobas® assay [24]) and ferritin, PCT and IL-6 were measured using Electro-Chemi Luminescent Immuno Assay (ECLIA) tests (E801 Cobas® assay [25]). Leukocyte counts, neutrophil counts, and lymphocyte counts were determined using Sysmex automated cell counters, and May-Grünwald/Giemsa staining and microscopic examination were employed for visual confirmation. LDH and ALAT levels were quantified through enzymatic analysis and albumin levels were measured using a colorimetric method with bromocresol purple (BCP).

**Table S1.** Luminex biomarkers with abbreviations and biomarker type

| **Biomarker** | **Abbreviation** | **Alias** | **Type of biomarker** | **Immune system involvement** |
| --- | --- | --- | --- | --- |
| Angiopoietin-2 | ANG-2 |  | Endothelial |  |
| Chemokine C-C motif ligand 2 | CCL2 | MCP-1/JE | Inflammation | Innate |
| Chemokine C-C motif ligand 5 | CCL5 | RANTES | Inflammation | Innate |
| Chemokine C-X-C motif ligand 8 | CXCL8 | IL-8 | Inflammation | Innate |
| Chemokine C-X-C motif ligand 10 | CXCL10 | IP-10/CRG-2 | Inflammation | Adaptive/Innate |
| Chemokine C-X-C motif ligand 16 | CXCL16 | SR-PSOX | Inflammation | Adaptive/Innate |
| Chemokine C-C motif ligand 20 | CCL20 | MIP-3α | Inflammation | Innate |
| Cluster of differentiation 163 | CD163 |  | Inflammation | Innate |
| Cluster of differentiation 40 ligand | CD40 Ligand | CD154/ TNFSF5 | Inflammation | Adaptive |
| Coagulation Factor III | CF3 | Tissue Factor | Coagulation |  |
| Complement Component C5a | C5a |  | Inflammation | Innate |
| Complement Component C9 | C9 |  | Inflammation | Innate |
| Epidermal growth factor | EGF |  | Growth factors |  |
| E-Selectin/CD62E | E-Selectin | CD62E | Endothelial/Fibroproliferative |  |
| Galectin-3 | LGALS3 | Mac-2 | Fibroproliferative | Innate |
| Granulocyte colony stimulating factor | G-CSF | CSF3 | Inflammation | Innate |
| Granulocyte macrophage-colony stimulating factor | GM-CSF | CSF2 | Inflammation | Innate |
| Intercellular Adhesion Molecule 1 | ICAM-1 | CD54 | Endothelial/Inflammation | Innate/Adaptive |
| Interferon alpha | IFNα |  | Inflammation | Innate |
| Interferon beta | IFNß |  | Inflammation | Innate |
| Interferon gamma | IFNγ |  | Inflammation | Innate |
| Interleukin 1 beta | IL-1β | IL-IF2 | Inflammation | Inflammasome |
| Interleukin 1 receptor antagonist | IL-1RA | IL-1F3 | Inflammation | Inflammasome |
| Interleukin 1 receptor inhibitor | IL-1RI |  | Inflammation | Innate |
| Interleukin 10 | IL-10 |  | Inflammation | Innate |
| Interleukin 17 | IL-17 | IL-17A | Inflammation | Adaptive |
| Interleukin 18 | IL-18 | IL-1F4 | Inflammation | Innate/Adaptive |
| Interleukin 28 | IL-28 | IFN-lambda 2 | Inflammation | Adaptive |
| Interleukin 2R alpha | IL-2RA | CD25 | Inflammation | Adaptive |
| Interleukin 33 | IL-33 |  | Inflammation | Inflammasome |
| Interleukin 4 | IL-4 |  | Inflammation | Adaptive |
| Interleukin 6 | IL-6 |  | Inflammation | Innate |
| Interleukin 6 receptor alpha | IL-6RA | CD126 | Inflammation | Innate |
| Interleukin 7 | IL-7 |  | Inflammation | Adaptive |
| Interleukin-12/Interleukin-23p40 | IL-12/IL-23p40 |  | Inflammation | Adaptive/Innate |
| Lactoferrin | LTF |  | Inflammation | Innate |
| Leptin | LEP | OB | Inflammation | Innate/Adaptive |
| Matrix metalloproteinase-9 | MMP-9 | Collagenase 1 | Fibroproliferative/Inflammation | Innate |
| Myeloperoxidase | MPO |  | Inflammation | Innate |
| Osteoprotegerin | OPG | Tnfrsf11b | Inflammation | Adaptive/Immune |
| Platelet endothelial cell adhesion molecule-1 | PECAM-1 | CD31 | Endothelial/Inflammation | Innate |
| Platelet-derived growth factor-BB | PDGF-BB |  | Growth factors/Fibroproliferative |  |
| P-Selectin | P-Selectin | CD62P | Coagulation |  |
| Receptor for advanced glycation endproducts | RAGE | AGER | Epithelial |  |
| Surfactant protein D | SP-D |  | Epithelial/Fibroproliferative |  |
| Thrombomodulin | THBD | BDCA-3 | Endothelial/Coagulation |  |
| Thrombopoietin | TPO |  | Coagulation |  |
| Tie-2 | Tie-2 | CD202b | Endothelial |  |
| Tissue factor pathway inhibitor | TFPI |  | Endothelial/Coagulation |  |
| Tumor necrosis factor alpha | TNFα |  | Inflammation | Innate |
| Urokinase-type plasminogen activator receptor | uPAR | CD87 | Fibroproliferative/inflammation | Innate |
| Vascular cell adhesion molecule 1 | VCAM-1 | CD106 | Endothelial |  |
| Vascular endothelial growth factor | VEGF |  | Growth factors/ Fibroproliferative/Endothelial |  |
| Von Willebrand factor-A2 | VWF-A2 |  | Coagulation/Endothelial |  |

**Table S1** presents the complete panel of Luminex biomarkers analyzed in this study. Biomarkers are categorized by type (inflammation, endothelial, fibroproliferative, epithelial, or growth factor) and immune system involvement (innate or adaptive). Abbreviations used throughout the manuscript correspond to those listed in this table and common aliases for these biomarkers are also provided.

**Table S2:** equivalent corticosteroids and dosages (total daily dosing)

| Drug | Equivalent dose (mg) | Low-dose (mg) | High-dose (mg) |
| --- | --- | --- | --- |
| Dexamethasone | 0.75 | ≤6 | >6 |
| Cortisone | 25 | ≤200 | >200 |
| Hydrocortisone | 20 | ≤160 | >160 |
| Prednis(ol)one  Methylprednisolone | 5  4 | ≤40  ≤32 | >40  >32 |
| Betamethasone | 0.75 | ≤6 | >6 |
| Table S2. This table shows the equivalent corticosteroids and dosages. Equivalent dose is derived from https://www.farmacotherapeutischkompas.nl/bladeren/groepsteksten/corticosteroiden__systemisch | | | |

**Table S3:** Treatment Protocol Stages for COVID-19 in the ICU at Erasmus Medical Center

| Stage | Immunomodulation | BAL | BAL Consequence | Notes |
| --- | --- | --- | --- | --- |
| I | Dexamethasone/Tocilizumab/ Sarilumab | No | Not applicable | • Dexamethasone 10 mg from start • No tocilizumab/sarilumab if <21 days already administered |
| IIA/B | MPS protocol (and tocilizumab/sarilumab) | Yes | Stop MPS only for: • H/VAP • CAPA  Not in case of colonization only | • No MPS initiation >14 days after diagnosis of moderate/severe ARDS, unless (see section "No MPS Protocol") |
| III | MPS protocol (and tocilizumab/sarilumab) | Yes | Do not stop MPS  Treat superinfection |  |

**Note**: Although in Dutch, the complete protocol is available as a separate supplementary file

**Table S4.** Luminex biomarker assay quality check

| **Biomarker** | **Within all limits** | **>ULQ** | **<LLQ** | **< 50 beads measured** |
| --- | --- | --- | --- | --- |
| ANG-2 | 99.8% | 1 (0.1%) | 0 (0.0%) | 1 (0.1%) |
| C5a | 99.9% | 0 (0.0%) | 0 (0.0%) | 1 (0.1%) |
| C9 | 99.9% | 0 (0.0%) | 0 (0.0%) | 1 (0.1%) |
| CCL2 | 99.9% | 0 (0.0%) | 0 (0.0%) | 1 (0.1%) |
| CCL20 | 99.2% | 4 (0.6%) | 1 (0.1%) | 1 (0.1%) |
| CCL5 | 99.9% | 0 (0.0%) | 0 (0.0%) | 1 (0.1%) |
| CD163 | 97.5% | 17 (2.5%) | 0 (0.0%) | 0 (0.0%) |
| CD31 | 99.9% | 0 (0.0%) | 0 (0.0%) | 1 (0.1%) |
| CD40 Ligand | 100.0% | 0 (0.0%) | 0 (0.0%) | 0 (0%) |
| CF3 | 100.0% | 0 (0.0%) | 0 (0.0%) | 0 (0%) |
| CXCL10 | 96.5% | 24 (3.5%) | 0 (0.0%) | 0 (0%) |
| CXCL16 | 98.1% | 13 (1.9%) | 0 (0.0%) | 0 (0%) |
| EGF | 99.8% | 0 (0.0%) | 1 (0.1%) | 1 (0.1%) |
| E-Selectin | 99.9% | 0 (0.0%) | 0 (0.0%) | 1 (0.1%) |
| Galectin-3 | 99.9% | 0 (0.0%) | 0 (0.0%) | 1 (0.1%) |
| G-CSF* | 96.1% | 1 (0.1%) | 0 (0.0%) | 26 (3.8%) |
| GM-CSF | 100.0% | 0 (0.0%) | 0 (0.0%) | 0 (0%) |
| ICAM-1 | 99.3% | 4 (0.6%) | 0 (0.0%) | 1 (0.1%) |
| IFN-α | 99.9% | 0 (0.0%) | 0 (0.0%) | 1 (0.1%) |
| IFN-β* | 65.8% | 0 (0.0%) | 237 (34.2%) | 0 (0.0%) |
| IFN-γ | 88.1% | 0 (0.0%) | 82 (11.8%) | 1 (0.1%) |
| IL-10 | 96.1% | 0 (0.0%) | 27 (3.9%) | 0 (0.0%) |
| IL-12p40 | 94.8% | 0 (0.0%) | 34 (4.9%) | 2 (0.3%) |
| IL-17 | 90.8% | 0 (0.0%) | 64 (9.2%) | 0 (0.0%) |
| IL-18 | 100.0% | 0 (0.0%) | 0 (0.0%) | 0 (0%) |
| IL-1RA | 96.1% | 27 (3.9%) | 0 (0.0%) | 0 (0%) |
| IL-1RI | 99.9% | 0 (0.0%) | 0 (0.0%) | 1 (0.1%) |
| IL-1β | 99.9% | 0 (0.0%) | 0 (0.0%) | 1 (0.1%) |
| IL-28 | 98.6% | 0 (0.0%) | 9 (1.3%) | 1 (0.1%) |
| IL-2RA | 99.9% | 0 (0.0%) | 0 (0.0%) | 1 (0.1%) |
| IL-33 | 90.9% | 0 (0.0%) | 62 (9.0%) | 1 (0.1%) |
| IL-4 | 99.9% | 0 (0.0%) | 0 (0.0%) | 1 (0.1%) |
| IL-6 | 98.6% | 9 (1.3%) | 0 (0.0%) | 1 (0.1%) |
| IL-6Rα | 100.0% | 0 (0.0%) | 0 (0.0%) | 0 (0.0%) |
| IL-7 | 100.0% | 0 (0.0%) | 0 (0.0%) | 0 (0.0%) |
| IL-8 | 99.9% | 0 (0.0%) | 0 (0.0%) | 1 (0.1%) |
| Lactoferrin | 99.0% | 6 (0.9%) | 0 (0.0%) | 1 (0.1%) |
| Leptin | 98.9% | 3 (0.4%) | 5 (0.7%) | 0 (0.0%) |
| MMP-9 | 99.8% | 1 (0.1%) | 0 (0.0%) | 1 (0.1%) |
| MPO | 99.9% | 0 (0.0%) | 0 (0.0%) | 1 (0.1%) |
| Osteoprotegerin | 99.7% | 0 (0.0%) | 0 (0.0%) | 2 (0.3%) |
| PDGF-BB | 99.8% | 0 (0.0%) | 1 (0.1%) | 1 (0.1%) |
| P-Selectin | 99.7% | 0 (0.0%) | 0 (0.0%) | 2 (0.3%) |
| RAGE | 99.9% | 0 (0.0%) | 0 (0.0%) | 1 (0.1%) |
| SP-D | 99.9% | 0 (0.0%) | 0 (0.0%) | 1 (0.1%) |
| TFPI | 99.9% | 0 (0.0%) | 0 (0.0%) | 1 (0.1%) |
| THBD | 99.9% | 0 (0.0%) | 0 (0.0%) | 1 (0.1%) |
| Tie-2 | 99.9% | 0 (0.0%) | 0 (0.0%) | 1 (0.1%) |
| TNF-α | 100.0% | 0 (0.0%) | 0 (0.0%) | 0 (0.0%) |
| TPO | 94.1% | 0 (0.0%) | 40 (5.8%) | 1 (0.1%) |
| uPAR | 99.9% | 0 (0.0%) | 0 (0.0%) | 1 (0.1%) |
| VCAM-1 | 98.4% | 11 (1.6%) | 0 (0.0%) | 0 (0.0%) |
| VEGF | 99.7% | 0 (0.0%) | 0 (0.0%) | 2 (0.3%) |
| vWF-A2 | 99.9% | 0 (0.0%) | 1 (0.1%) | 0 (0.0%) |

**Table S4.** Data quality was assessed by evaluation of beads count (the number of replicates of a biomarker concentration measured). Measurements with a bead count lower than 50 were considered a too low quality and were excluded. Values below the lowest point of the calibration curve were imputed with the lowest value. Samples above the highest point of the calibration curve were imputed with the highest value. *Biomarkers were considered unreliable and therefore excluded from the analyses.

**Table S5.** Statistical analysis of significant biomarker changes over time

| Biomarker | Sum Sq | Mean Sq | NumDF | DenDF | F-value | p-value |
| --- | --- | --- | --- | --- | --- | --- |
| RAGE | 22.43 | 11.22 | 2 | 82.07 | 51.53 | <0.001 |
| IL-6 | 85.91 | 42.95 | 2 | 36.82 | 41.72 | <0.001 |
| CXCL10 | 49.45 | 24.73 | 2 | 67.94 | 27.83 | <0.001 |
| VEGF | 21.82 | 10.91 | 2 | 87.02 | 21.20 | <0.001 |
| CF3 | 2.13 | 1.06 | 2 | 81.49 | 20.14 | <0.001 |
| IL-18 | 3.36 | 1.68 | 2 | 88.29 | 17.48 | <0.001 |
| VCAM-1 | 10.47 | 5.23 | 2 | 108.69 | 15.81 | <0.001 |
| GM-CSF | 5.10 | 2.55 | 2 | 51.04 | 18.39 | <0.001 |
| Lactoferrin | 26.62 | 13.31 | 2 | 51.98 | 14.94 | <0.001 |
| CCL2 | 23.96 | 11.98 | 2 | 51.62 | 14.84 | <0.001 |
| Galectin-3 | 5.05 | 2.52 | 2 | 44.87 | 14.72 | <0.001 |
| IL-6 (standard care) | 42.10 | 21.05 | 2 | 46.62 | 14.09 | <0.001 |
| CRP | 30.43 | 15.22 | 2 | 85.31 | 12.42 | <0.001 |
| Ferritin | 3.16 | 1.58 | 2 | 92.76 | 12.19 | <0.001 |
| Leukocyte count | 3.72 | 1.86 | 2 | 68.53 | 12.41 | <0.001 |
| IL-10 | 18.56 | 9.28 | 2 | 73.84 | 11.69 | <0.001 |
| IL-7 | 1.88 | 0.94 | 2 | 57.20 | 11.70 | <0.001 |
| ALAT | 7.83 | 3.92 | 2 | 91.74 | 10.70 | <0.001 |
| TNF-α | 3.20 | 1.60 | 2 | 74.76 | 10.28 | <0.001 |
| Osteoprotegerin | 5.25 | 2.62 | 2 | 52.11 | 9.28 | <0.001 |
| IL-6Rα | 1.16 | 0.58 | 2 | 138.43 | 8.00 | <0.001 |
| LDH | 1.15 | 0.58 | 2 | 67.24 | 8.00 | <0.001 |
| CXCL16 | 1.57 | 0.79 | 2 | 72.70 | 7.91 | <0.001 |
| C5a | 4.77 | 2.39 | 2 | 84.22 | 7.65 | <0.001 |
| NLR | 5.00 | 2.50 | 2 | 51.26 | 7.79 | <0.01 |
| IL-33 | 9.41 | 4.71 | 2 | 90.25 | 5.36 | <0.01 |
| Albumin | 0.38 | 0.19 | 2 | 65.23 | 5.15 | <0.01 |
| IFN-α | 6.16 | 3.08 | 2 | 58.25 | 5.14 | <0.01 |
| D-dimer | 5.33 | 2.66 | 2 | 73.48 | 5.04 | <0.01 |
| IL-28 | 3.28 | 1.64 | 2 | 86.13 | 4.50 | 0.01 |
| Procalcitonin | 6.48 | 3.24 | 2 | 102.99 | 4.29 | 0.02 |
| Tie-2 | 0.95 | 0.47 | 2 | 121.56 | 4.09 | 0.02 |
| CCL20 | 6.86 | 3.43 | 2 | 45.11 | 4.29 | 0.02 |
| IL-1RI | 0.41 | 0.21 | 2 | 84.08 | 4.10 | 0.02 |
| Leptin | 9.82 | 4.91 | 2 | 54.56 | 4.16 | 0.02 |
| MMP-9 | 7.38 | 3.69 | 2 | 62.45 | 4.09 | 0.02 |
| IL-8 | 3.56 | 1.78 | 2 | 67.56 | 3.73 | 0.03 |
| E-Selectin | 0.55 | 0.27 | 2 | 89.74 | 3.33 | 0.04 |
| IFN-γ | 0.89 | 0.45 | 2 | 67.58 | 3.27 | 0.04 |

**Table S5.** Statistical analysis of significant biomarker changes over time. Abbreviations: ALAT: Alanine Aminotransferase; CRP: C-Reactive Protein; DenDF: denominator degrees of freedom; F-value: F-test statistic; LDH: Lactate Dehydrogenase; Mean Sq: mean sum of squares; NLR: Neutrophil-to-Lymphocyte Ratio; NumDF: numerator degrees of freedom; p-value: probability value; Sum Sq: sum of squares

**Table S6.** HRs and adjusted p-values for the significant biomarkers

| Biomarker | HR | Age | Gender | BMI | HDS | *P*-value biomarker | Direction mortality risk | |
| --- | --- | --- | --- | --- | --- | --- | --- | --- |
| Albumin (standard care) | 0.16 (0.043-0.59) | 1.02 | 0.33 | 0.99 | 3.27 | 0.021 | | **↓** |
| Angiopoietin-2 | 2.27 (1.63-3.23) | 1.02 | 0.23 | 0.98 | 5.6 | <0.0001 | | **↑** |
| CD31/PECAM | 2.90 (1.24-7.04) | 1.04 | 0.24 | 0.98 | 3.32 | 0.043 | | **↑** |
| Chemokine C-C motif ligand 20 | 1.75 (1.41-2.17) | 1.02 | 0.34 | 0.97 | 4.16 | <0.0001 | | **↑** |
| Chemokine C-C motif ligand 5 | 0.67 (0.49-0.90) | 1.01 | 0.21 | 0.99 | 3.08 | 0.03 | | **↓** |
| Chemokine C-X-C motif ligand 16 | 2.66 (1.54-4.46) | 1.01 | 0.16 | 0.97 | 2.72 | 0.0036 | | **↑** |
| Cluster of differentiation 163 | 1.60 (1.18-2.17) | 1.02 | 0.31 | 0.97 | 3.61 | 0.012 | | **↑** |
| CRP (standard care) | 1.45 (1.13-192) | 1.02 | 0.29 | 0.98 | 4.42 | 0.012 | | **↑** |
| D-dimer (standard care) | 1.42 (1.08-1.90) | 1.03 | 0.25 | 1 | 4.47 | 0.043 | | **↑** |
| E-Selectin | 3.78 (2.25-6.52) | 1.04 | 0.39 | 1 | 4.76 | <0.0001 | | **↑** |
| Galectin-3 | 1.91 (1.14-3.15) | 1.02 | 0.23 | 0.96 | 4.09 | 0.048 | | **↑** |
| Granulocyte macrophage-colony stimulating factor sstimulatingstifactor | 1.89 (1.17-3.13) | 1.02 | 0.24 | 0.97 | 3.46 | 0.029 | | **↑** |
| Intercellular Adhesion Molecule 1 | 2.28 (1.27-4.19) | 1.03 | 0.25 | 0.98 | 4.69 | 0.028 | | **↑** |
| Interleukin 1β | 3.84 (2.00-7.79) | 1.04 | 0.34 | 0.96 | 2.67 | <0.0001 | | **↑** |
| Interleukin 2R alpha | 1.55 (1.10-2.19) | 1.03 | 0.24 | 0.99 | 3.94 | 0.037 | | **↑** |
| Interleukin 4 | 8.56 (2.93-27.46) | 1.03 | 0.34 | 0.97 | 2.62 | 0.0021 | | **↑** |
| Interleukin 6 | 1.35 (1.08-1.69) | 1.02 | 0.28 | 0.98 | 3.98 | 0.027 | | **↑** |
| Interleukin 6 (standard care) | 1.32 (1.10-1.60) | 1.03 | 0.3 | 1 | 4.19 | 0.016 | | **↑** |
| Lactoferin | 1.50 (1.06-2.11) | 1.04 | 0.25 | 0.98 | 5.43 | 0.048 | | **↑** |
| MPO | 1.63 (1.16-2.28) | 1.03 | 0.29 | 0.96 | 4.37 | 0.023 | | **↑** |
| NLR (standard care) | 1.74 (1.20-2.58) | 1 | 0.35 | 0.96 | 2.62 | 0.021 | | **↑** |
| Osteoprotegerin | 2.69 (1.62-4.51) | 1 | 0.33 | 0.97 | 3.12 | <0.0001 | | **↑** |
| Procalcitonin (standard care) | 1.26 (1.09-1.47) | 1.02 | 0.28 | 0.98 | 3.89 | 0.012 | | **↑** |
| Surfactant protein D | 1.52 (1.19-1.94) | 1.01 | 0.21 | 0.97 | 3.4 | 0.0064 | | **↑** |
| Urokinase-type plasminogen activator receptor | 1.85 (1.40-2.47) | 1.03 | 0.31 | 0.93 | 3.61 | <0.0001 | | **↑** |
| VEGF | 0.67 (0.47-0.94) | 1.03 | 0.22 | 0.99 | 3.17 | 0.048 | | **↓** |
| Table S6. In this table the HRs and p-values after correction for multiple testing using the False Discovery Rate method are presented. Abbreviations: HR, Hazard Ratio; BMI, Body Mass Index; HDS, High-Dose Corticosteroids | | | | | | | | |

**Table S7.** Hazard ratios of all biomarkers for ICU mortality risk

| Biomarker |  | HR | 2.5% | 97.5% | P-value |
| --- | --- | --- | --- | --- | --- |
| CCL5 | Age | 1.01 | 0.97 | 1.05 | 0.77 |
| CCL5 | Sex (male) | 0.21 | 0.05 | 0.72 | 0.01 |
| CCL5 | BMI | 0.99 | 0.93 | 1.06 | 0.84 |
| CCL5 | HDS | 3.08 | 1.64 | 5.9 | 0 |
| CCL5 | Log2(CCL5) | 0.67 | 0.49 | 0.9 | 0.01 |
| LGALS3 | Age | 1.02 | 0.98 | 1.07 | 0.28 |
| LGALS3 | Sex (male) | 0.23 | 0.05 | 0.76 | 0.01 |
| LGALS3 | BMI | 0.96 | 0.9 | 1.02 | 0.21 |
| LGALS3 | HDS | 4.09 | 2.26 | 7.71 | 0 |
| LGALS3 | log2(LGALS3) | 1.91 | 1.14 | 3.15 | 0.02 |
| MMP-9 | Age | 1.03 | 0.99 | 1.07 | 0.13 |
| MMP-9 | Sex (male) | 0.24 | 0.05 | 0.82 | 0.02 |
| MMP-9 | BMI | 0.98 | 0.91 | 1.04 | 0.49 |
| MMP-9 | HDS | 3.89 | 2.12 | 7.16 | 0 |
| MMP-9 | log2(MMP-9) | 0.74 | 0.48 | 1.15 | 0.18 |
| PDGF-BB | Age | 1.02 | 0.98 | 1.06 | 0.44 |
| PDGF-BB | Sex (male) | 0.19 | 0.04 | 0.65 | 0.01 |
| PDGF-BB | BMI | 0.99 | 0.92 | 1.05 | 0.69 |
| PDGF-BB | HDS | 3.27 | 1.81 | 6.13 | 0 |
| PDGF-BB | log2(PDGF-BB) | 0.77 | 0.6 | 0.99 | 0.04 |
| C9 | Age | 1.04 | 1 | 1.08 | 0.06 |
| C9 | Sex (male) | 0.26 | 0.06 | 0.89 | 0.03 |
| C9 | BMI | 0.97 | 0.9 | 1.03 | 0.34 |
| C9 | HDS | 3.7 | 2.06 | 6.83 | 0 |
| C9 | log2(C9) | 1.81 | 0.7 | 5.17 | 0.23 |
| LTF | Age | 1.04 | 1 | 1.08 | 0.05 |
| LTF | Sex (male) | 0.25 | 0.05 | 0.8 | 0.02 |
| LTF | BMI | 0.98 | 0.91 | 1.04 | 0.48 |
| LTF | HDS | 5.43 | 2.64 | 11.65 | 0 |
| LTF | log2(LTF) | 1.5 | 1.06 | 2.11 | 0.02 |
| MPO | Age | 1.03 | 0.99 | 1.08 | 0.11 |
| MPO | Sex (male) | 0.29 | 0.06 | 1.01 | 0.05 |
| MPO | BMI | 0.96 | 0.9 | 1.03 | 0.3 |
| MPO | HDS | 4.37 | 2.33 | 8.37 | 0 |
| MPO | log2(MPO) | 1.63 | 1.16 | 2.28 | 0.01 |
| vWF-A2 | Age | 1.03 | 1 | 1.08 | 0.09 |
| vWF-A2 | Sex (male) | 0.25 | 0.06 | 0.84 | 0.02 |
| vWF-A2 | BMI | 0.97 | 0.91 | 1.04 | 0.41 |
| vWF-A2 | HDS | 3.61 | 1.99 | 6.82 | 0 |
| vWF-A2 | log2(vWF-A2) | 1.04 | 0.64 | 1.68 | 0.87 |
| CCL2 | Age | 1.03 | 0.99 | 1.07 | 0.21 |
| CCL2 | Sex (male) | 0.25 | 0.06 | 0.85 | 0.02 |
| CCL2 | BMI | 0.97 | 0.91 | 1.04 | 0.42 |
| CCL2 | HDS | 3.43 | 1.89 | 6.4 | 0 |
| CCL2 | log2(CCL2) | 1.24 | 0.9 | 1.67 | 0.18 |
| IL-1β | Age | 1.04 | 1 | 1.08 | 0.06 |
| IL-1β | Sex (male) | 0.34 | 0.07 | 1.16 | 0.09 |
| IL-1β | BMI | 0.96 | 0.9 | 1.03 | 0.27 |
| IL-1β | HDS | 2.67 | 1.44 | 5.09 | 0 |
| IL-1β | log2(IL-1β) | 3.84 | 2 | 7.79 | 0 |
| IL-4 | Age | 1.03 | 0.99 | 1.07 | 0.22 |
| IL-4 | Sex (male) | 0.34 | 0.07 | 1.17 | 0.09 |
| IL-4 | BMI | 0.96 | 0.9 | 1.03 | 0.3 |
| IL-4 | HDS | 2.62 | 1.38 | 5.06 | 0 |
| IL-4 | log2(IL-4) | 8.56 | 2.93 | 27.46 | 0 |
| IL-12 | Age | 1.03 | 0.99 | 1.08 | 0.1 |
| IL-12 | Sex (male) | 0.23 | 0.05 | 0.8 | 0.02 |
| IL-12 | BMI | 0.97 | 0.91 | 1.04 | 0.4 |
| IL-12 | HDS | 3.8 | 2.04 | 7.08 | 0 |
| IL-12 | log2(IL-12) | 0.96 | 0.72 | 1.36 | 0.78 |
| E-Selectin | Age | 1.04 | 1.01 | 1.09 | 0.02 |
| E-Selectin | Sex (male) | 0.39 | 0.08 | 1.35 | 0.16 |
| E-Selectin | BMI | 1 | 0.93 | 1.07 | 0.95 |
| E-Selectin | HDS | 4.76 | 2.49 | 9.38 | 0 |
| E-Selectin | log2(E-Selectin) | 3.78 | 2.25 | 6.52 | 0 |
| TFPI | Age | 1.03 | 0.99 | 1.08 | 0.11 |
| TFPI | Sex (male) | 0.25 | 0.06 | 0.83 | 0.02 |
| TFPI | BMI | 0.97 | 0.91 | 1.04 | 0.37 |
| TFPI | HDS | 3.52 | 1.94 | 6.62 | 0 |
| TFPI | log2(TFPI) | 1.1 | 0.82 | 1.52 | 0.56 |
| Tie-2 | Age | 1.03 | 0.99 | 1.08 | 0.11 |
| Tie-2 | Sex (male) | 0.25 | 0.06 | 0.84 | 0.02 |
| Tie-2 | BMI | 0.97 | 0.91 | 1.04 | 0.4 |
| Tie-2 | HDS | 3.66 | 2.05 | 6.91 | 0 |
| Tie-2 | log2(Tie-2) | 1.03 | 0.71 | 1.59 | 0.91 |
| VEGF | Age | 1.02 | 0.99 | 1.07 | 0.22 |
| VEGF | Sex (male) | 0.22 | 0.05 | 0.73 | 0.01 |
| VEGF | BMI | 0.99 | 0.93 | 1.05 | 0.72 |
| VEGF | HDS | 3.17 | 1.75 | 5.84 | 0 |
| VEGF | log2(VEGF) | 0.67 | 0.47 | 0.94 | 0.02 |
| PECAM-1 | Age | 1.04 | 1 | 1.08 | 0.05 |
| PECAM-1 | Sex (male) | 0.24 | 0.05 | 0.77 | 0.01 |
| PECAM-1 | BMI | 0.98 | 0.91 | 1.04 | 0.45 |
| PECAM-1 | HDS | 3.32 | 1.8 | 6.13 | 0 |
| PECAM-1 | log2(PECAM-1) | 2.9 | 1.24 | 7.04 | 0.01 |
| IL-1 RI | Age | 1.03 | 0.99 | 1.07 | 0.11 |
| IL-1 RI | Sex (male) | 0.25 | 0.05 | 0.84 | 0.02 |
| IL-1 RI | BMI | 0.97 | 0.9 | 1.03 | 0.39 |
| IL-1 RI | HDS | 3.71 | 2.07 | 6.94 | 0 |
| IL-1 RI | log2(IL-1 RI) | 0.78 | 0.39 | 1.57 | 0.48 |
| CXCL8 | Age | 1.03 | 0.99 | 1.08 | 0.12 |
| CXCL8 | Sex (male) | 0.29 | 0.06 | 0.96 | 0.04 |
| CXCL8 | BMI | 0.97 | 0.91 | 1.03 | 0.35 |
| CXCL8 | HDS | 3 | 1.64 | 5.72 | 0 |
| CXCL8 | log2(CXCL8) | 1.35 | 1.04 | 1.76 | 0.03 |
| OPG | Age | 1 | 0.96 | 1.05 | 0.96 |
| OPG | Sex (male) | 0.32 | 0.07 | 1.16 | 0.09 |
| OPG | BMI | 0.97 | 0.9 | 1.04 | 0.4 |
| OPG | HDS | 3.15 | 1.72 | 6.06 | 0 |
| OPG | log2(OPG) | 2.69 | 1.62 | 4.51 | 0 |
| SP-D | Age | 1.03 | 0.99 | 1.07 | 0.2 |
| SP-D | Sex (male) | 0.21 | 0.05 | 0.69 | 0.01 |
| SP-D | BMI | 0.97 | 0.91 | 1.04 | 0.45 |
| SP-D | HDS | 3.4 | 1.91 | 6.39 | 0 |
| SP-D | log2(SP-D) | 1.52 | 1.19 | 1.94 | 0 |
| THBD | Age | 1.03 | 0.99 | 1.07 | 0.23 |
| THBD | Sex (male) | 0.27 | 0.06 | 0.93 | 0.04 |
| THBD | BMI | 0.96 | 0.9 | 1.02 | 0.24 |
| THBD | HDS | 3.29 | 1.82 | 6.23 | 0 |
| THBD | log2(THBD) | 1.49 | 0.9 | 2.48 | 0.12 |
| uPAR | Age | 1.03 | 0.98 | 1.07 | 0.27 |
| uPAR | Sex (male) | 0.31 | 0.07 | 1.05 | 0.06 |
| uPAR | BMI | 0.93 | 0.86 | 1 | 0.05 |
| uPAR | HDS | 3.61 | 1.93 | 6.81 | 0 |
| uPAR | log2(uPAR) | 1.86 | 1.4 | 2.47 | 0 |
| ANG-2 | Age | 1.02 | 0.97 | 1.06 | 0.49 |
| ANG-2 | Sex (male) | 0.23 | 0.05 | 0.79 | 0.02 |
| ANG-2 | BMI | 0.98 | 0.91 | 1.05 | 0.6 |
| ANG-2 | HDS | 6 | 3.06 | 12.09 | 0 |
| ANG-2 | log2(ANG-2) | 2.28 | 1.63 | 3.23 | 0 |
| CD40 Ligand | Age | 1.03 | 1 | 1.08 | 0.09 |
| CD40 Ligand | Sex (male) | 0.25 | 0.05 | 0.83 | 0.02 |
| CD40 Ligand | BMI | 0.98 | 0.91 | 1.04 | 0.5 |
| CD40 Ligand | HDS | 3.61 | 2.02 | 6.68 | 0 |
| CD40 Ligand | log2(CD40 Ligand) | 1.06 | 0.68 | 1.58 | 0.76 |
| IL-2RA | Age | 1.03 | 0.99 | 1.07 | 0.16 |
| IL-2RA | Sex (male) | 0.24 | 0.05 | 0.8 | 0.02 |
| IL-2RA | BMI | 0.99 | 0.93 | 1.06 | 0.88 |
| IL-2RA | HDS | 3.94 | 2.18 | 7.28 | 0 |
| IL-2RA | log2(IL-2RA) | 1.55 | 1.1 | 2.19 | 0.01 |
| C5a | Age | 1.03 | 1 | 1.08 | 0.09 |
| C5a | Sex (male) | 0.24 | 0.05 | 0.85 | 0.02 |
| C5a | BMI | 0.97 | 0.91 | 1.04 | 0.44 |
| C5a | HDS | 3.65 | 2 | 6.88 | 0 |
| C5a | log2(C5a) | 0.98 | 0.75 | 1.31 | 0.87 |
| CXCL16 | Age | 1.01 | 0.97 | 1.06 | 0.54 |
| CXCL16 | Sex (male) | 0.16 | 0.03 | 0.56 | 0 |
| CXCL16 | BMI | 0.97 | 0.91 | 1.04 | 0.4 |
| CXCL16 | HDS | 2.72 | 1.46 | 5.17 | 0 |
| CXCL16 | log2(CXCL16) | 2.66 | 1.54 | 4.46 | 0 |
| G-CSF | Age | 1.04 | 1 | 1.08 | 0.08 |
| G-CSF | Sex (male) | 0.26 | 0.06 | 0.85 | 0.03 |
| G-CSF | BMI | 0.97 | 0.91 | 1.04 | 0.43 |
| G-CSF | HDS | 3.44 | 1.89 | 6.48 | 0 |
| G-CSF | log2(G-CSF) | 1.26 | 0.63 | 2.5 | 0.5 |
| ICAM-1 | Age | 1.04 | 1 | 1.08 | 0.05 |
| ICAM-1 | Sex (male) | 0.25 | 0.05 | 0.81 | 0.01 |
| ICAM-1 | BMI | 0.98 | 0.92 | 1.05 | 0.55 |
| ICAM-1 | HDS | 4.69 | 2.49 | 9.26 | 0 |
| ICAM-1 | log2(ICAM-1) | 2.28 | 1.27 | 4.19 | 0.01 |
| IFNß | Age | 1.03 | 0.99 | 1.08 | 0.09 |
| IFNß | Sex (male) | 0.24 | 0.05 | 0.84 | 0.02 |
| IFNß | BMI | 0.98 | 0.91 | 1.04 | 0.49 |
| IFNß | HDS | 3.76 | 2.08 | 7 | 0 |
| IFNß | log2(IFNß) | 0.94 | 0.85 | 1.04 | 0.25 |
| IL-1RA | Age | 1.02 | 0.99 | 1.07 | 0.23 |
| IL-1RA | Sex (male) | 0.28 | 0.06 | 0.93 | 0.04 |
| IL-1RA | BMI | 0.96 | 0.89 | 1.02 | 0.21 |
| IL-1RA | HDS | 3.44 | 1.91 | 6.44 | 0 |
| IL-1RA | log2(IL-1RA) | 1.22 | 1.02 | 1.45 | 0.03 |
| IL-7 | Age | 1.03 | 1 | 1.08 | 0.1 |
| IL-7 | Sex (male) | 0.26 | 0.06 | 0.9 | 0.03 |
| IL-7 | BMI | 0.97 | 0.91 | 1.04 | 0.4 |
| IL-7 | HDS | 3.98 | 2.16 | 7.72 | 0 |
| IL-7 | log2(IL-7) | 1.59 | 0.66 | 3.87 | 0.27 |
| IL-17 | Age | 1.03 | 1 | 1.08 | 0.09 |
| IL-17 | Sex (male) | 0.25 | 0.05 | 0.84 | 0.02 |
| IL-17 | BMI | 0.97 | 0.91 | 1.04 | 0.43 |
| IL-17 | HDS | 3.65 | 2.05 | 6.79 | 0 |
| IL-17 | log2(IL-17) | 1 | 0.89 | 1.13 | 0.94 |
| IL-28 | Age | 1.03 | 1 | 1.08 | 0.08 |
| IL-28 | Sex (male) | 0.26 | 0.06 | 0.88 | 0.03 |
| IL-28 | BMI | 0.98 | 0.91 | 1.04 | 0.46 |
| IL-28 | HDS | 3.68 | 2.08 | 6.94 | 0 |
| IL-28 | log2(IL-28) | 0.96 | 0.82 | 1.15 | 0.61 |
| IL-6RA | Age | 1.03 | 0.99 | 1.07 | 0.17 |
| IL-6RA | Sex (male) | 0.29 | 0.06 | 0.98 | 0.05 |
| IL-6RA | BMI | 0.97 | 0.9 | 1.03 | 0.3 |
| IL-6RA | HDS | 3.59 | 1.99 | 6.66 | 0 |
| IL-6RA | log2(IL-6RA) | 1.59 | 1.02 | 2.88 | 0.03 |
| RAGE | Age | 1.04 | 1 | 1.08 | 0.07 |
| RAGE | Sex (male) | 0.24 | 0.05 | 0.79 | 0.02 |
| RAGE | BMI | 0.98 | 0.91 | 1.04 | 0.48 |
| RAGE | HDS | 3.38 | 1.87 | 6.22 | 0 |
| RAGE | log2(RAGE) | 0.87 | 0.67 | 1.12 | 0.27 |
| TPO | Age | 1.03 | 1 | 1.07 | 0.09 |
| TPO | Sex (male) | 0.25 | 0.05 | 0.84 | 0.02 |
| TPO | BMI | 0.97 | 0.91 | 1.04 | 0.39 |
| TPO | HDS | 3.63 | 2.02 | 6.72 | 0 |
| TPO | log2(TPO) | 1.02 | 0.88 | 1.2 | 0.88 |
| VCAM-1 | Age | 1.03 | 0.99 | 1.07 | 0.16 |
| VCAM-1 | Sex (male) | 0.28 | 0.06 | 0.94 | 0.04 |
| VCAM-1 | BMI | 0.97 | 0.91 | 1.03 | 0.37 |
| VCAM-1 | HDS | 3.32 | 1.71 | 6.48 | 0 |
| VCAM-1 | log2(VCAM-1) | 1.1 | 0.88 | 1.38 | 0.43 |
| CCL20 | Age | 1.02 | 0.98 | 1.07 | 0.27 |
| CCL20 | Sex (male) | 0.34 | 0.07 | 1.18 | 0.1 |
| CCL20 | BMI | 0.97 | 0.9 | 1.04 | 0.4 |
| CCL20 | HDS | 4.16 | 2.26 | 7.98 | 0 |
| CCL20 | log2(CCL20) | 1.6 | 1.3 | 1.97 | 0 |
| CD163 | Age | 1.02 | 0.98 | 1.07 | 0.35 |
| CD163 | Sex (male) | 0.31 | 0.07 | 1.05 | 0.06 |
| CD163 | BMI | 0.97 | 0.91 | 1.04 | 0.39 |
| CD163 | HDS | 3.61 | 2.03 | 6.63 | 0 |
| CD163 | log2(CD163) | 1.6 | 1.18 | 2.17 | 0 |
| CF3 | Age | 1.02 | 0.99 | 1.07 | 0.25 |
| CF3 | Sex (male) | 0.24 | 0.05 | 0.81 | 0.02 |
| CF3 | BMI | 0.97 | 0.91 | 1.03 | 0.34 |
| CF3 | HDS | 3.53 | 1.97 | 6.51 | 0 |
| CF3 | log2(CF3) | 1.64 | 0.89 | 3.03 | 0.12 |
| CXCL10 | Age | 1.02 | 0.98 | 1.07 | 0.3 |
| CXCL10 | Sex (male) | 0.3 | 0.07 | 1.01 | 0.05 |
| CXCL10 | BMI | 0.97 | 0.9 | 1.03 | 0.36 |
| CXCL10 | HDS | 3.29 | 1.77 | 6.2 | 0 |
| CXCL10 | log2(CXCL10) | 1.23 | 0.98 | 1.52 | 0.07 |
| EGF | Age | 1.02 | 0.98 | 1.07 | 0.33 |
| EGF | Sex (male) | 0.24 | 0.05 | 0.8 | 0.02 |
| EGF | BMI | 0.98 | 0.92 | 1.05 | 0.57 |
| EGF | HDS | 3.44 | 1.85 | 6.38 | 0 |
| EGF | log2(EGF) | 0.82 | 0.63 | 1.07 | 0.13 |
| GM-CSF | Age | 1.02 | 0.98 | 1.07 | 0.3 |
| GM-CSF | Sex (male) | 0.23 | 0.05 | 0.76 | 0.02 |
| GM-CSF | BMI | 0.97 | 0.9 | 1.03 | 0.32 |
| GM-CSF | HDS | 3.46 | 1.93 | 6.41 | 0 |
| GM-CSF | log2(GM-CSF) | 1.89 | 1.17 | 3.13 | 0.01 |
| IFNα | Age | 1.03 | 0.99 | 1.08 | 0.1 |
| IFNα | Sex (male) | 0.25 | 0.06 | 0.83 | 0.02 |
| IFNα | BMI | 0.98 | 0.91 | 1.04 | 0.49 |
| IFNα | HDS | 3.63 | 2.04 | 6.71 | 0 |
| IFNα | log2(IFNα) | 0.94 | 0.68 | 1.28 | 0.7 |
| IFNγ | Age | 1.03 | 1 | 1.08 | 0.1 |
| IFNγ | Sex (male) | 0.25 | 0.05 | 0.85 | 0.02 |
| IFNγ | BMI | 0.97 | 0.91 | 1.04 | 0.42 |
| IFNγ | HDS | 3.6 | 1.98 | 6.68 | 0 |
| IFNγ | log2(IFNγ) | 0.97 | 0.7 | 1.42 | 0.86 |
| IL-6 | Age | 1.02 | 0.99 | 1.07 | 0.26 |
| IL-6 | Sex (male) | 0.28 | 0.06 | 0.95 | 0.04 |
| IL-6 | BMI | 0.98 | 0.91 | 1.05 | 0.64 |
| IL-6 | HDS | 3.98 | 2.18 | 7.56 | 0 |
| IL-6 | log2(IL-6) | 1.35 | 1.08 | 1.69 | 0.01 |
| IL-10 | Age | 1.03 | 1 | 1.08 | 0.09 |
| IL-10 | Sex (male) | 0.25 | 0.05 | 0.88 | 0.02 |
| IL-10 | BMI | 0.97 | 0.91 | 1.03 | 0.4 |
| IL-10 | HDS | 3.57 | 1.99 | 6.57 | 0 |
| IL-10 | log2(IL-10) | 1.04 | 0.84 | 1.34 | 0.77 |
| IL-18 | Age | 1.03 | 0.99 | 1.07 | 0.1 |
| IL-18 | Sex (male) | 0.25 | 0.06 | 0.82 | 0.02 |
| IL-18 | BMI | 0.97 | 0.91 | 1.04 | 0.41 |
| IL-18 | HDS | 3.63 | 2.04 | 6.71 | 0 |
| IL-18 | log2(IL-18) | 1 | 0.69 | 1.44 | 0.99 |
| IL-33 | Age | 1.03 | 0.99 | 1.08 | 0.1 |
| IL-33 | Sex (male) | 0.24 | 0.05 | 0.82 | 0.02 |
| IL-33 | BMI | 0.97 | 0.91 | 1.03 | 0.42 |
| IL-33 | HDS | 3.65 | 2.04 | 6.76 | 0 |
| IL-33 | log2(IL-33) | 0.98 | 0.84 | 1.17 | 0.76 |
| LEP | Age | 1.04 | 1 | 1.08 | 0.07 |
| LEP | Sex (male) | 0.27 | 0.06 | 0.91 | 0.03 |
| LEP | BMI | 0.99 | 0.91 | 1.08 | 0.9 |
| LEP | HDS | 3.77 | 2.08 | 6.97 | 0 |
| LEP | log2(LEP) | 0.91 | 0.72 | 1.14 | 0.46 |
| P-Selectin | Age | 1.05 | 1.01 | 1.1 | 0.02 |
| P-Selectin | Sex (male) | 0.26 | 0.05 | 0.88 | 0.02 |
| P-Selectin | BMI | 0.97 | 0.9 | 1.04 | 0.41 |
| P-Selectin | HDS | 4.75 | 2.39 | 9.61 | 0 |
| P-Selectin | log2(P-Selectin) | 0.97 | 0.84 | 1.02 | 0.73 |
| TNF-α | Age | 1.03 | 0.99 | 1.07 | 0.21 |
| TNF-α | Sex (male) | 0.29 | 0.06 | 0.93 | 0.03 |
| TNF-α | BMI | 0.97 | 0.9 | 1.04 | 0.38 |
| TNF-α | HDS | 3.81 | 2.12 | 7.08 | 0 |
| TNF-α | log2(TNFα) | 1.52 | 0.96 | 2.41 | 0.07 |
| D-dimer | Age | 1.03 | 0.99 | 1.07 | 0.13 |
| D-dimer | Sex (male) | 0.25 | 0.05 | 0.85 | 0.02 |
| D-dimer | BMI | 1 | 0.93 | 1.07 | 0.97 |
| D-dimer | HDS | 4.47 | 2.37 | 8.66 | 0 |
| D-dimer | log2(d-dimer) | 1.42 | 1.08 | 1.9 | 0.02 |
| LDH | Age | 1.03 | 0.99 | 1.07 | 0.15 |
| LDH | Sex (male) | 0.25 | 0.05 | 0.88 | 0.03 |
| LDH | BMI | 0.97 | 0.91 | 1.04 | 0.41 |
| LDH | HDS | 3.54 | 1.94 | 6.61 | 0 |
| LDH | log2(LDH) | 2.02 | 0.9 | 4.26 | 0.08 |
| CRP | Age | 1.02 | 0.98 | 1.07 | 0.28 |
| CRP | Sex (male) | 0.29 | 0.06 | 1 | 0.05 |
| CRP | BMI | 0.98 | 0.92 | 1.05 | 0.63 |
| CRP | HDS | 4.42 | 2.38 | 8.52 | 0 |
| CRP | log2(CRP) | 1.45 | 1.13 | 1.92 | 0 |
| Ferritin | Age | 1.03 | 0.99 | 1.07 | 0.1 |
| Ferritin | Sex (male) | 0.26 | 0.05 | 0.87 | 0.03 |
| Ferritin | BMI | 0.97 | 0.91 | 1.04 | 0.41 |
| Ferritin | HDS | 3.66 | 2.03 | 6.95 | 0 |
| Ferritin | log2(ferritin) | 1.07 | 0.86 | 1.34 | 0.52 |
| Procalcitonin | Age | 1.02 | 0.98 | 1.06 | 0.36 |
| Procalcitonin | Sex (male) | 0.28 | 0.06 | 0.94 | 0.04 |
| Procalcitonin | BMI | 0.98 | 0.91 | 1.04 | 0.49 |
| Procalcitonin | HDS | 3.89 | 2.14 | 7.3 | 0 |
| Procalcitonin | log2(procalcitonin) | 1.26 | 1.09 | 1.47 | 0 |
| ALAT | Age | 1.03 | 0.99 | 1.08 | 0.1 |
| ALAT | Sex (male) | 0.24 | 0.06 | 0.82 | 0.02 |
| ALAT | BMI | 0.97 | 0.91 | 1.04 | 0.44 |
| ALAT | HDS | 3.65 | 2.04 | 6.76 | 0 |
| ALAT | log2(ALAT) | 0.95 | 0.72 | 1.26 | 0.73 |
| IL-6 (standard care) | Age | 1.03 | 0.99 | 1.07 | 0.18 |
| IL-6 (standard care) | Sex (male) | 0.3 | 0.06 | 1 | 0.05 |
| IL-6 (standard care) | BMI | 1 | 0.93 | 1.07 | 0.95 |
| IL-6 (standard care) | HDS | 4.19 | 2.32 | 8.02 | 0 |
| IL-6 (standard care) | log2(IL-6) | 1.32 | 1.1 | 1.6 | 0 |
| Albumin | Age | 1.02 | 0.98 | 1.06 | 0.46 |
| Albumin | Sex (male) | 0.32 | 0.07 | 1.15 | 0.09 |
| Albumin | BMI | 0.99 | 0.92 | 1.06 | 0.72 |
| Albumin | HDS | 3.27 | 1.82 | 6.07 | 0 |
| Albumin | log2(albumin) | 0.16 | 0.04 | 0.59 | 0 |
| Leukocyte count | Age | 1.03 | 0.99 | 1.07 | 0.14 |
| Leukocyte count | Sex (male) | 0.27 | 0.05 | 0.89 | 0.03 |
| Leukocyte count | BMI | 0.97 | 0.9 | 1.04 | 0.35 |
| Leukocyte count | HDS | 3.71 | 2.03 | 7.07 | 0 |
| Leukocyte count | log2(leukocyte count) | 1.87 | 1.04 | 3.45 | 0.03 |
| NLR | Age | 1 | 0.97 | 1.05 | 0.86 |
| NLR | Sex (male) | 0.35 | 0.08 | 1.15 | 0.09 |
| NLR | BMI | 0.96 | 0.89 | 1.02 | 0.17 |
| NLR | HDS | 2.62 | 1.3 | 5.28 | 0.01 |
| NLR | log2(NLR) | 1.74 | 1.2 | 2.58 | 0 |

**Table S7.** In this table the HRs of all biomarkers are shown adjusted for age, gender, BMI and HDS. A Log2 transformation was applied and the reported HR’s to express the relative change in the risk of ICU death resulting from a doubling of the biomarker’s value in a day compared to no change in the same period.

| Gene Ontology ID | Description | % | Gene-ID’s | Background Ratio | Fold Enrichment | FDR |
| --- | --- | --- | --- | --- | --- | --- |
| GO:0030335 | positive regulation of cell migration | 34.8 | IL4, CSF2, CCL20, CCL5, IL1B, PECAM1, VEGFA, CXCL16 | 287/19734 | 23.9 | 0.0000096 |
| GO:0007159 | leukocyte cell-cell adhesion | 21.7 | CALCA, CCL5, PECAM1, SELE, ICAM1 | 32/19734 | 134.1 | 0.0000109 |
| GO:0002548 | monocyte chemotaxis | 21.7 | LGALS3, IL6, CALCA, CCL20, CCL5 | 47/19734 | 91.3 | 0.0000334 |
| GO:0006954 | inflammatory response | 34.8 | CRP, IL6, CALCA, CCL20, CCL5, IL1B, IL2RA, SELE | 422/19734 | 16.3 | 0.0000334 |
| GO:0048246 | macrophage chemotaxis | 17.4 | LGALS3, CCL5, IL1B, SFTPD | 17/19734 | 201.9 | 0.0000861 |
| GO:0006955 | immune response | 30.4 | IL4, CSF2, CCL20, CCL5, IL1B, IL2RA, PECAM1 | 505/19734 | 11.9 | 0.0012723 |
| GO:0042102 | positive regulation of T cell proliferation | 17.4 | IL4, IL6, CCL5, IL1B | 61/19734 | 56.3 | 0.0031542 |
| GO:0042531 | positive regulation of tyrosine phosphorylation of STAT protein | 17.4 | IL4, IL6, CSF2, CCL5 | 67/19734 | 51.2 | 0.0036578 |
| GO:0030593 | neutrophil chemotaxis | 17.4 | LGALS3, CCL20, CCL5, IL1B | 83/19734 | 41.3 | 0.0061644 |
| GO:0045779 | negative regulation of bone resorption | 13.0 | IL6, CALCA, TNFRSF11B | 18/19734 | 143.0 | 0.0090848 |
| GO:0043066 | negative regulation of apoptotic process | 26.1 | IL4, IL6, PLAUR, MPO, LTF, VEGFA | 507/19734 | 10.2 | 0.0090848 |
| GO:0010628 | positive regulation of gene expression | 26.1 | CRP, IL4, IL6, CSF2, IL1B, VEGFA | 509/19734 | 10.1 | 0.0090848 |
| GO:0006935 | chemotaxis | 17.4 | CCL20, CCL5, PLAUR, CXCL16 | 121/19734 | 28.4 | 0.0130142 |
| GO:0034612 | response to tumor necrosis factor | 13.0 | CCL5, SELE, CXCL16 | 31/19734 | 83.0 | 0.0205571 |
| GO:0045671 | negative regulation of osteoclast differentiation | 13.0 | IL4, CALCA, TNFRSF11B | 33/19734 | 78.0 | 0.0217567 |
| GO:0006953 | acute-phase response | 13.0 | CRP, IL6, CD163 | 40/19734 | 64.4 | 0.0299899 |
| GO:0050796 | regulation of insulin secretion | 13.0 | IL6, CCL5, IL1B | 43/19734 | 59.9 | 0.0326107 |
| GO:0050918 | positive chemotaxis | 13.0 | LGALS3, CCL5, VEGFA | 50/19734 | 51.5 | 0.0415847 |
| GO:0001934 | positive regulation of protein phosphorylation | 17.4 | IL1B, PLAUR, PECAM1, VEGFA | 206/19734 | 16.7 | 0.0417553 |

**Table S8:** biological processes associated with IC-mortality

**Table S8.** Gene ontology analysis of differentially expressed biomarkers was performed using DAVID (Database for Annotation, Visualization and Integrated Discovery). The table shows the enriched biological processes with their associated Gene Ontology ID, description of the biological process, percentage of genes from the input list involved in the process, involved genes, background ratio (number of genes associated with the term in the database/total number of genes in the database), fold enrichment (indicating the magnitude of enrichment compared to expected frequency), and False Discovery Rate (FDR). Processes with a FDR < 0.05 were considered significant.

| **Table S9.** Demographic and clinical characteristics of patients categorized by high-dose corticosteroid treatment | | | |
| --- | --- | --- | --- |
|  | **No high-dose corticosteroids N = 110** | **High-dose corticosteroids   N= 52** | **P value** |
| **Patients’ characteristics** |  |  |  |
| Male, (N, %) | 81/110 (74%) | 44/52 (85%) | 0.20 |
| Age in years | 65 (56-71) | 63 (59-70) | 0.70 |
| BMI in kg/m^2^ | 29.3 (26.2-32.4) | 27.8 (25.0-32.3) | 0.40 |
| Current smoking (N, %) | 2 (4.2%) | 0 (0%) | 0.016 |
| Days from hospital admission to ICU admission | 0 (0-0) | 0 (0-0) | >0.99 |
| **Comorbidities, (N, %)** |  |  |  |
| Hypertension | 40/86 (47%) | 11/30 (37%) | 0.50 |
| Peripheral vascular disease | 5/88 (5.7%) | 0/30 (0%) | 0.30 |
| CVA/TIA | 5/88 (5.7%) | 1/30 (3.3%) | >0.99 |
| Chronic pulmonary disease | 9/85 (11%) | 2/29 (6.9%) | 0.70 |
| Chronic kidney disease | 5/88 (5.7%) | 4/30 (13%) | 0.20 |
| Immunodeficiency | 6/86 (7%) | 3/30 (10%) | 0.70 |
| Malignancies | 7/88 (8%) | 3/30 (10%) | 0.70 |
| Diabetes mellitus | 18/88 (20%) | 5/30 (17%) | 0.80 |
| **Admission characteristics** |  |  |  |
| APACHE IV predicted mortality score | 0.17 (0.11, 0.33) | 0.24 (0.13, 0.32) | 0.5 |
| SOFA score | 6 (3-9) | 7 (5-9) | 0.3 |
| PF-ratio, kPa | 29 (20, 37) | 26 (19, 34) | 0.3 |
| C reactive protein, mg/L | 144 (18, 315) | 40 (10, 219) | 0.1 |
| D-dimer, mg/L | 1.8 (1.0, 3.8) | 1.6 (1.0, 4.0) | 0.7 |
| Lactate dehydrogenase, U/L | 323 (269, 408) | 346 (292, 425) | 0.2 |
| Ferritin, ug/L | 1,275 (781, 1,889) | 1,244 (765, 1,735) | 0.9 |
| Procalcitonin, ng/mL | 0.3 (0.1, 1.1) | 0.4 (0.2, 1.5) | 0.7 |
| Alanine aminotransferase, U/L | 45 (27, 78) | 58 (30, 87) | 0.2 |
| Interleukin-6, pg/L | 123 (51, 292) | 120 (19, 269) | 0.6 |
| Albumin, g/L | 21.0 (18.0, 24.0) | 21.0 (17.0, 23.0) | 0.7 |
| **Table 2 Continued** |  |  |  |
| Leukocytes, ×10^9^ | 9.5 (7.3, 11.9) | 10.1 (7.6, 13.1) | 0.5 |
| Neutrophil/Leukocyte ratio | 7 (4, 11) | 13 (9, 22) | <0.001 |
| **Medication administered during admission, (N, %)** |  |  |  |
| Tociluzimab | 26 (24%) | 14 (27%) | 0.8 |
| Dexamethason 6mg** | 43 (39%) | 33 (63%) | 0.005 |
| **Clinical outcomes** |  |  |  |
| ICU length of stay in days | 14 (8-24) | 24 (14-32) | <0.001 |
| ICU mortality | 19 (17%) | 24 (46 | <0.001 |

**Table S9.** All continuous variables are reported as median [IQR] and all categorical variables as counts and percentages. High-dose corticosteroid (HDS) therapy was defined as treatment with >6 mg of dexamethasone per day or equivalent as indicated during ARDS. Patients treated with HDS and patients not treated with HDS were compared using a Wilcoxon test for continuous variables and Pearson's χ2 test for categorical variables. There were missing data for BMI in 2 patients (1.2%), for SOFA score in 11 patients (6.8%), for P/F ratio in 16 patients (9.9%) and for APACHE-IV score in 40 patients (24.7%). Abbreviations: APACHE, acute physiology, age, and chronic health evaluation; ARDS, acute respiratory distress syndrome; BMI, body mass index; FiO₂, fraction of inspired oxygen; PaO₂, arterial oxygen pressure; SOFA, sequential organ failure assessment

**Supplemental figures**

**Figure S1.** Swimmer plot of 162 patients included with sample availability

**
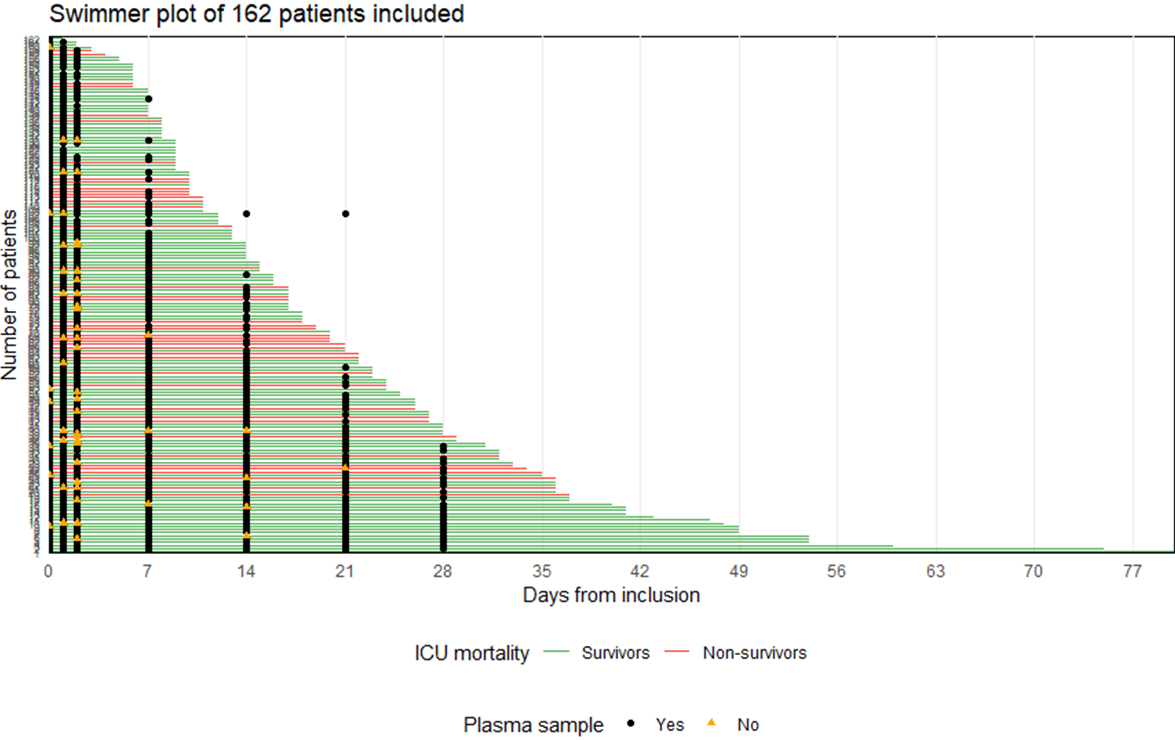
Figure S1.** Swimmer plot showing the timeline and sampling of 162 patients included in the study. Each horizontal line represents an unique patient, from the time of study inclusion to either ICU discharge or death. Dots indicate the availability of plasma samples, while orange triangles represent the absence of a sample. The color of the line indicates the patient’s vital status at ICU discharge. * One patient was sampled on day 14 and 21 when already discharged from the ICU and admitted to the general ward.

**Figure S2. Kaplan-Meier curve of survival**


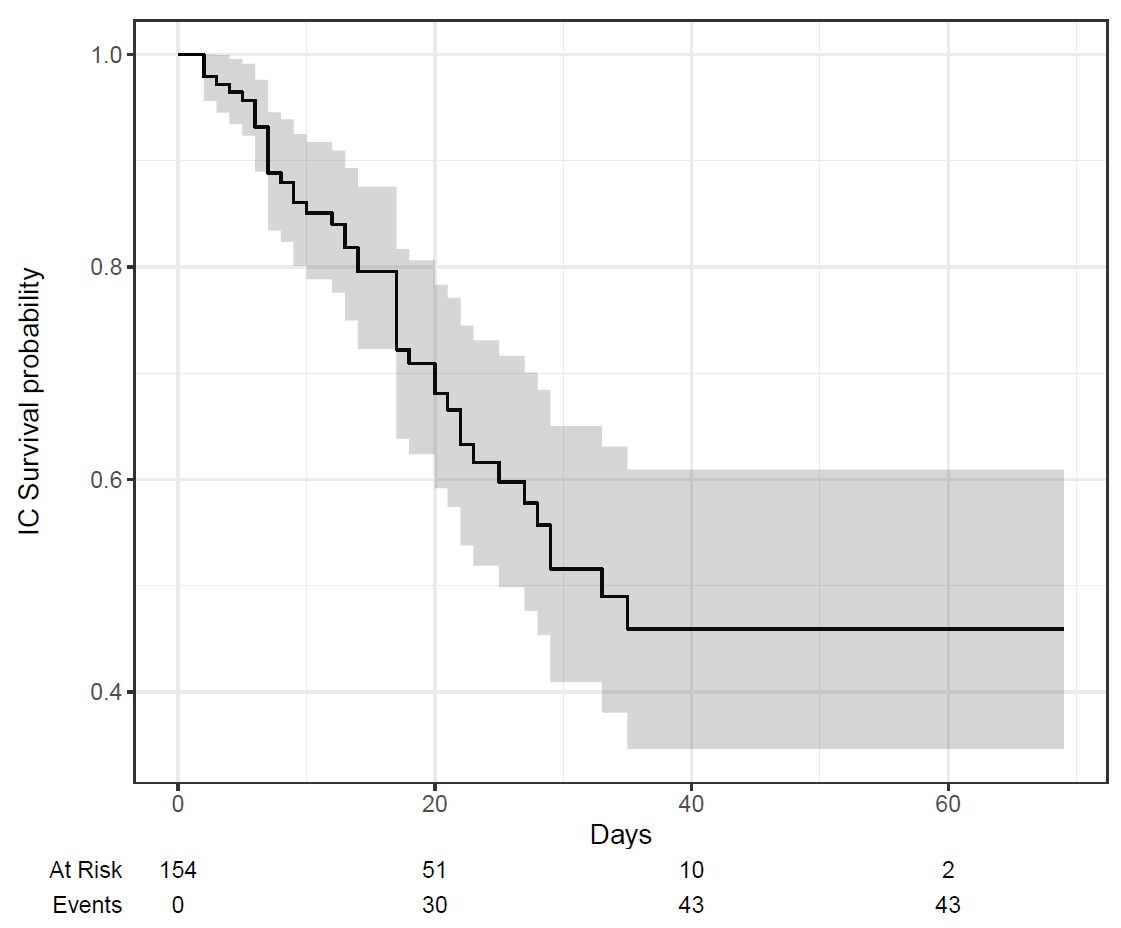


**Figure S2.** On the x-axis, time from ICU admission is displayed in days, and on the y-axis, the probability of survival in the ICU is presented. Patients are censored at ICU discharge Abbreviations; IC, intensive care.

**Figure S3.** Trajectories of biomarkers significantly associated with ICU mortality stratified by survival status.


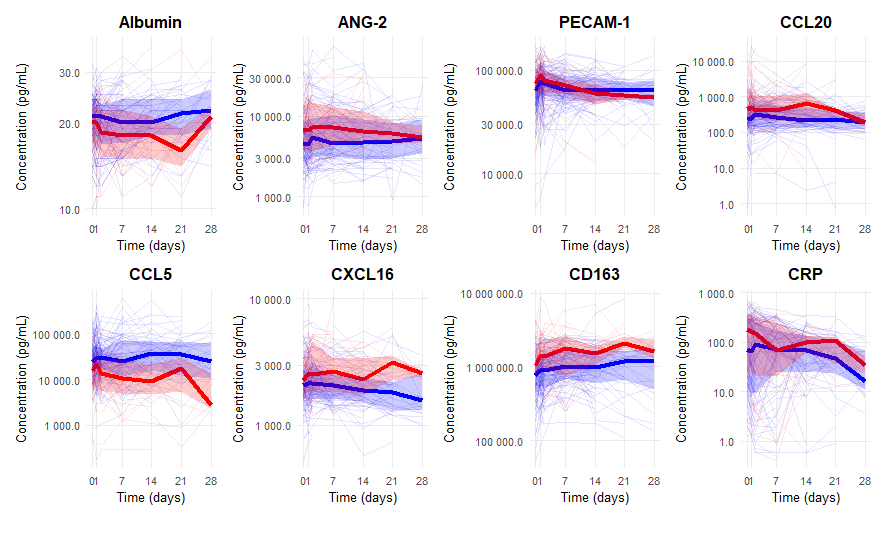


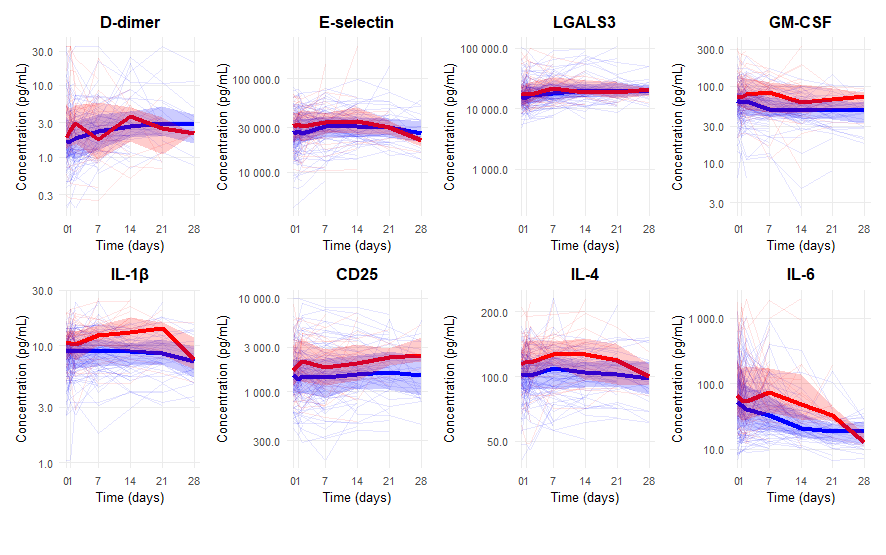


**
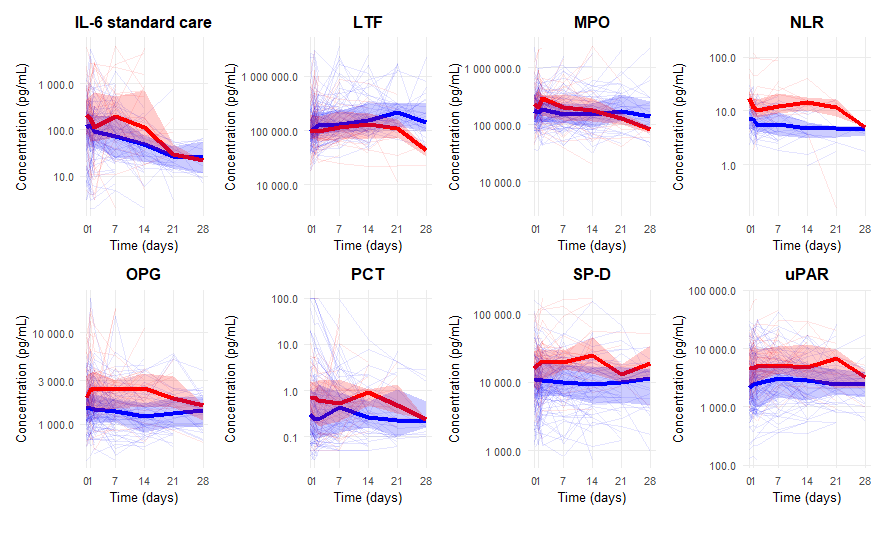
**

**
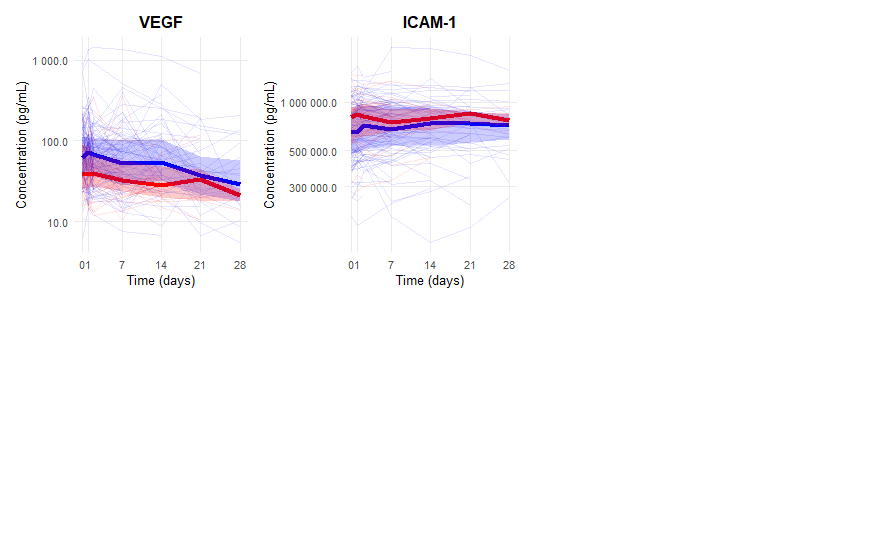
**

**Figure S3**. Individual biomarker trajectories of ICU patients over 28 days with median values (bold lines) and interquartile ranges (shaded areas) stratified by ICU outcome (blue = survived, red = died). The y-axis is displayed on a logarithmic scale. Measurements were taken at days 0, 1, 7, 14, 21 and 28 after ICU admission. Missing values per biomarker (total number): NLR (301), IL-6 standard care (129), D-dimer (134), PCT (111), Albumin (46), CRP (46), CD163 (17), CXCL16 (13), LTF (6), CCL20 (5), ICAM-1 (4), IL-6 research (9), ANG-2 (1).

**Figure S4**. STRING network visualization of biomarkers significantly associated with ICU mortality

**
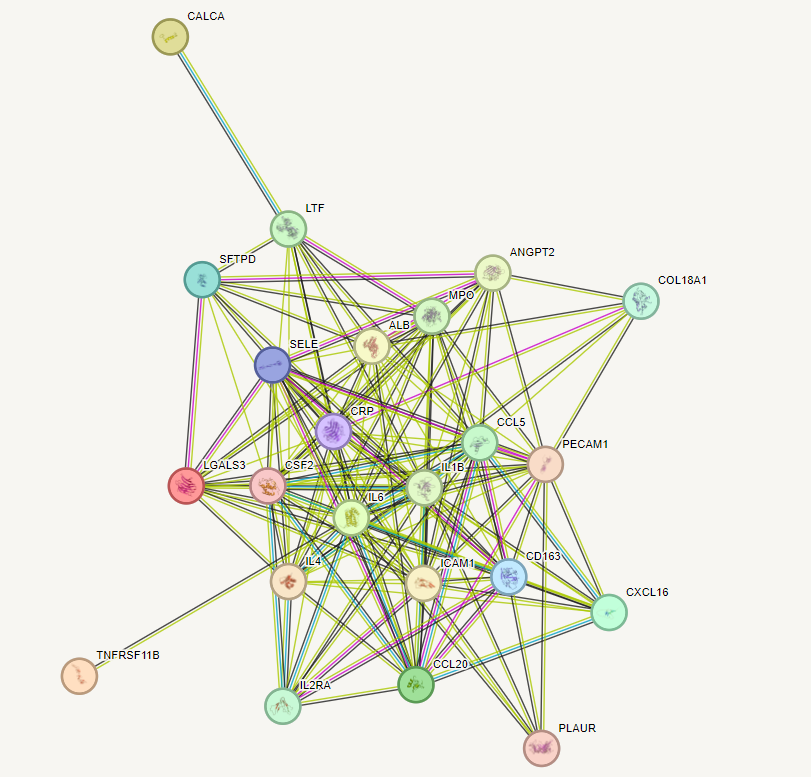

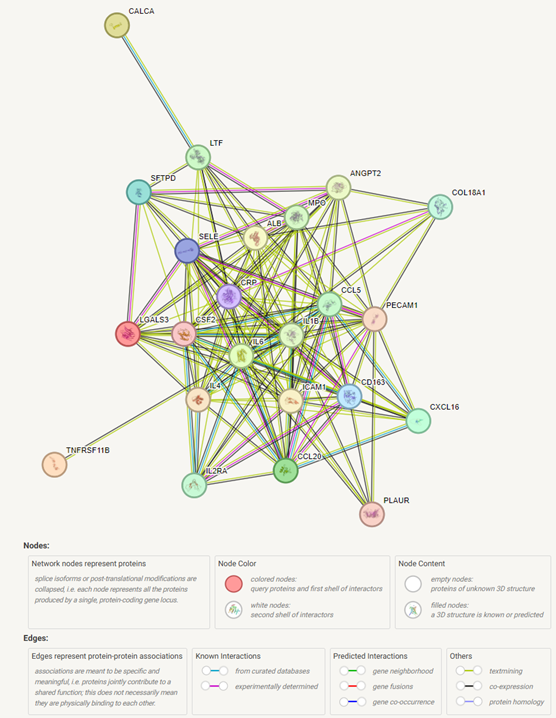
**

**Figure S4.** Protein–protein interaction network generated using the STRING database, illustrating interconnections between biomarkers significantly associated with ICU mortality. Each node represents a protein, and each edge represents a protein–protein association. The network was constructed using a minimum required interaction score of 0.4 (medium confidence). Node colors indicate distinct proteins or protein clusters, and edge thickness reflects the strength of supporting evidence for each interaction. Edge colors denote the type of evidence: cyan for curated database information, pink for experimentally determined interactions, green for gene neighborhood, red for gene fusions, and blue for gene co-occurrence. Additional associations include text mining (light brown), co-expression (black), and protein homology (light blue).

**Figure S5**. Cytoscape network visualization of biomarkers significantly associated with ICU mortality


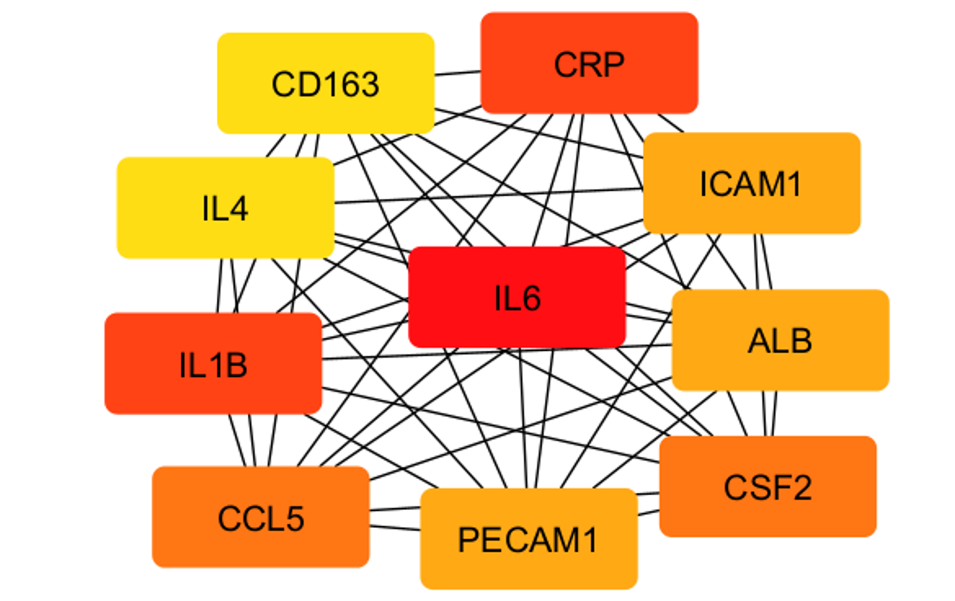


**Figure S5.** Protein interaction network generated using Cytoscape[1], displaying the top 10 most central biomarkers from all significantly associated biomarkers with ICU mortality. The proteins are represented by rectangles, with colors indicating their relative importance in the network (red indicating higher centrality). The connecting lines show protein-protein interactions, where the presence of a line indicates a known or predicted functional association between proteins.


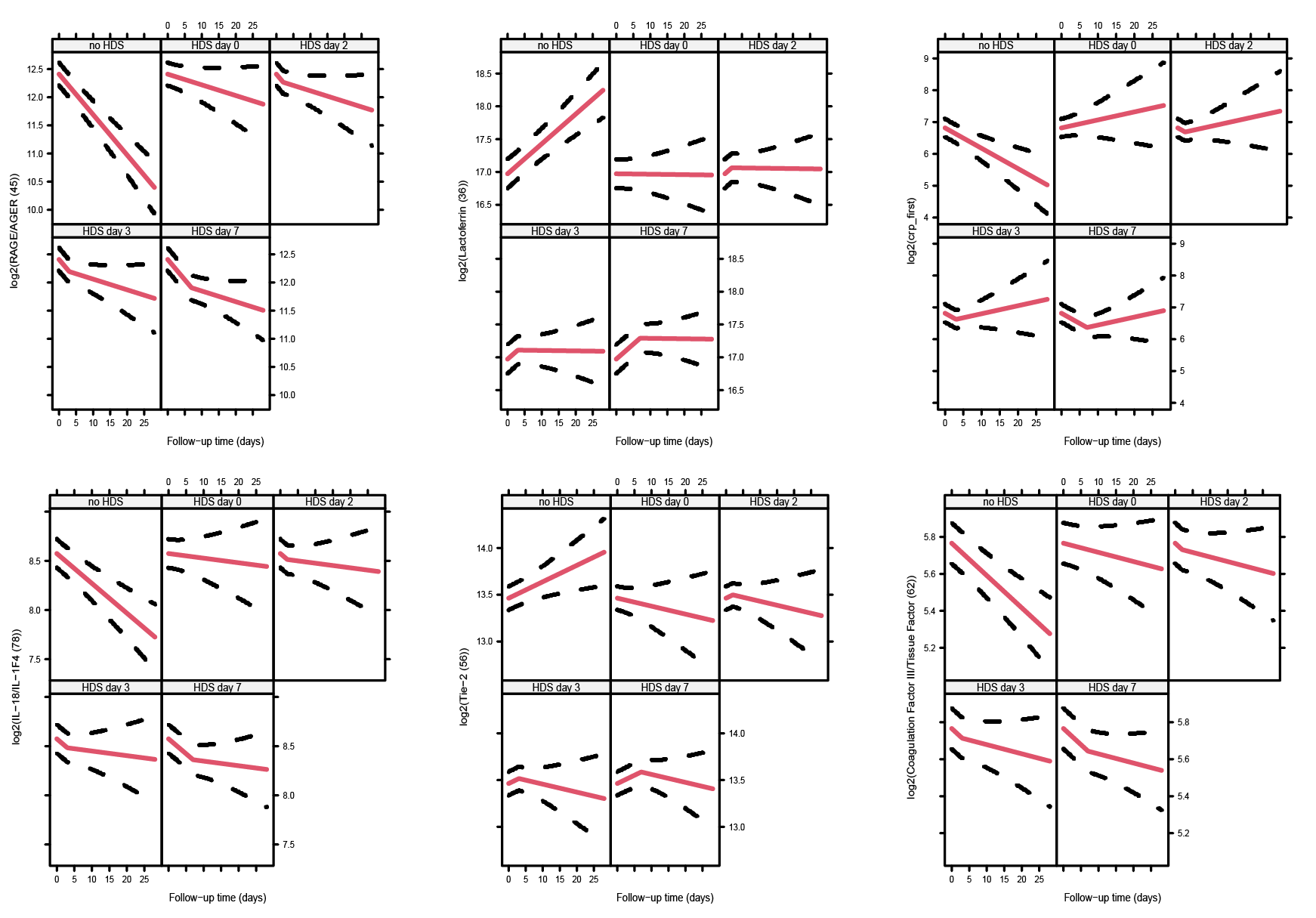


**Figure S5.** Effect of HDS on biomarker trajectories


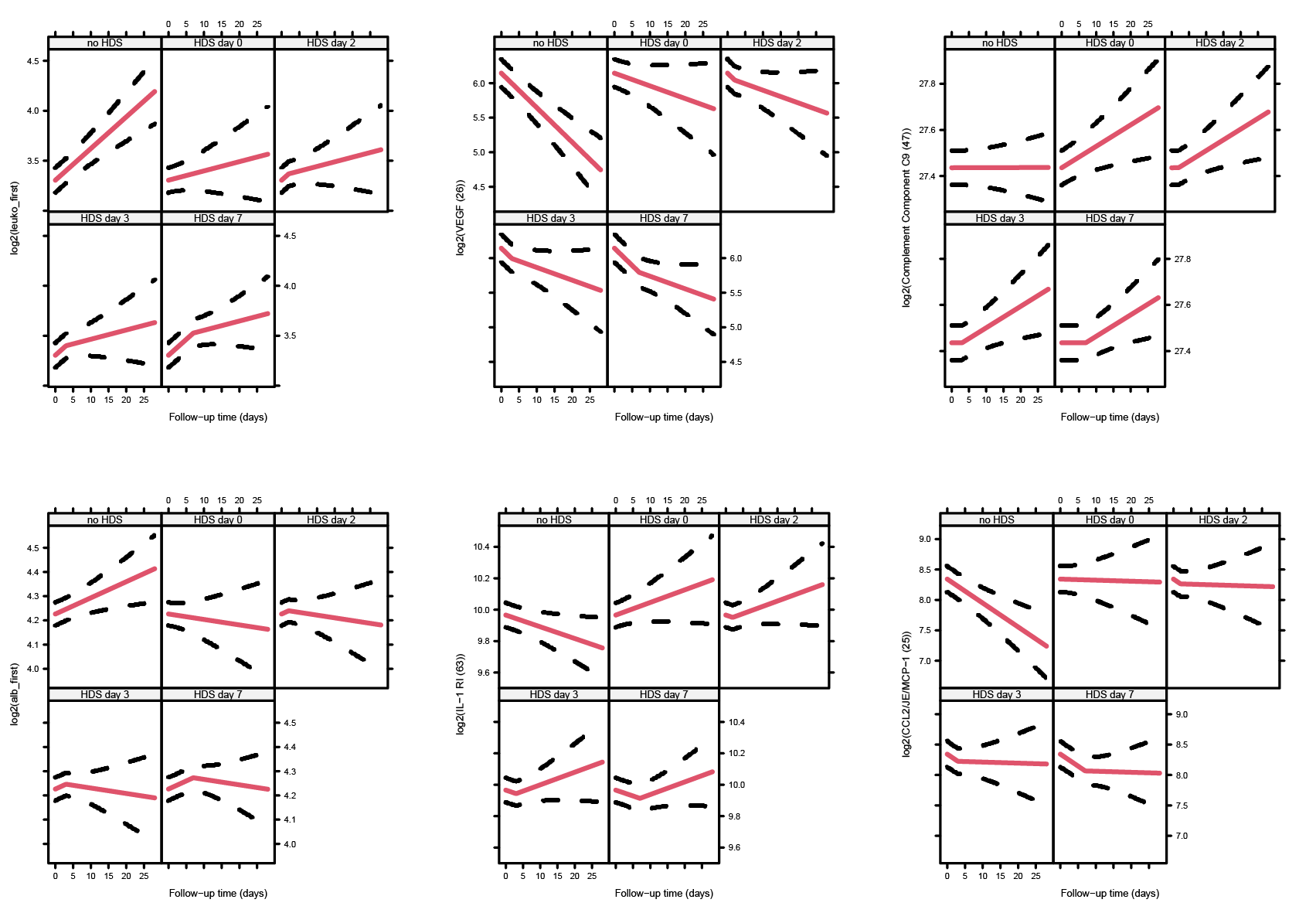


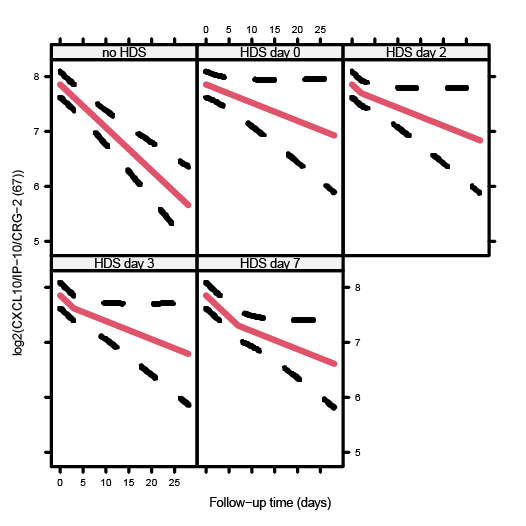


**Figure 5.** Trajectories of biomarker levels that were significantly different between patients treated with HDS (n=48) and without HDS therapy (n=114). The figure shows the trajectory of log₂-transformed biomarker levels (y-axis) versus follow-up time in days (x-axis) for different time points. Red lines represent the mean predicted values with 95% confidence intervals shown as dashed black lines. The panels display the temporal changes in biomarkers when not treated with HDS, at HDS initiation at day 0, and at subsequent timepoints days 2, 3, and 7.
